# Fluorene- and fluorenone-based molecules as electron-transporting SAMs for photovoltaic devices†

**DOI:** 10.1039/d4ra00964a

**Published:** 2024-05-10

**Authors:** Lauryna Monika Svirskaite, Ernestas Kasparavičius, Matas Steponaitis, Raitis Grzibovskis, Marius Franckevičius, Atanas Katerski, Arnas Naujokaitis, Smagul Karazhanov, Sajeesh Vadakkedath Gopi, Arturs Aizstrauts, Aivars Vembris, Vytautas Getautis, Tadas Malinauskas

**Affiliations:** a Department of Organic Chemistry, Kaunas University of Technology Radvilenu pl. 19 Kaunas 50254 Lithuania tadas.malinauskas@ktu.lt; b Department of Material and Environmental Technology, Tallinn University of Technology Ehitajate tee 5 Tallinn 19086 Estonia; c Department for Solar Energy, Institute for Energy Technology PO BOX 40 2027 Kjeller Norway; d Institute of Solid State Physics, University of Latvia Kengaraga st. 8 Riga LV-1063 Latvia; e Center for Physical Sciences and Technology Sauletekio Ave. 3 10257 Vilnius Lithuania

## Abstract

New semiconductors containing fluorene or fluorenone central fragments along with phosphonic acid anchoring groups were synthesized and investigated as electron transporting materials for possible application in photovoltaic devices. These derivatives demonstrate good thermal stability and suitable electrochemical properties for effective electron transport from perovskite, Sb_2_S_3_ and Sb_2_Se_3_ absorber layers. Self-assembled fluorene and fluorenone electron-transporting materials have shown improved substrate wettability, indicating bond formation between monolayer-forming compounds and the ITO, TiO_2_, Sb_2_S_3_, or Sb_2_Se_3_ surface. Additionally, investigated materials have compatible energetic band alignment and can passivate perovskite interface defects, which makes them interesting candidates for application in the n-i-p structure perovskite solar cell.

## Abstract 2

New semiconductors containing fluorene or fluorenone central fragments along with phosphonic acid anchoring groups were synthesized and investigated as electron transporting materials for possible application in photovoltaic devices.

## Graphical abstract

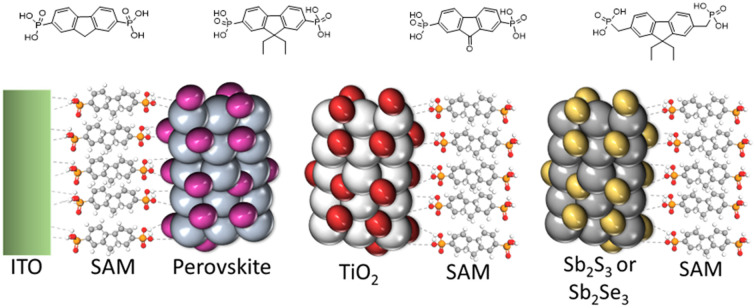

## Introduction

Due to its high power conversion efficiency and well-established production processes, silicon solar cell technology dominates the photovoltaic industry worldwide, occupying a large market share of the current world production (97% in 2022).^1^ However, the relatively high production cost of monocrystalline silicon and low optical absorption coefficient (*α* ≈ 100 cm^−1^) due to its indirect energy bandgap (≈1.1 eV) at room temperature are the main limitations for these solar cells.^2^ Next-generation devices based on perovskite, metal sulfides or metal selenides are not yet commercially viable; however, they demonstrate numerous evident advantages over silicon solar cells, *e.g.*, lower manufacturing cost, more efficient operation at low illumination, and suitability for semi-transparent or flexible devices.^2–4^

In the last decade, non-toxic and widespread Sb_2_Se_3_ and Sb_2_S_3_ have been developed as very appealing and promising light-absorbing materials for photovoltaics.^5^ Sb_2_Se_3_ and Sb_2_S_3_ layers demonstrate long-term stability and suitable optoelectronic properties, *e.g.*, a high absorption coefficient (*α* > 105 cm^−1^ and *α* > 104 cm^−1−*z*^, respectively)^6,7^ and narrow optical bandgap (1.1 eV and 1.1–1.3 eV, respectively),^8^ which approach the ideal Shockley–Queisser value.^9^ Furthermore, these chalcogenides can be used as a very thin layer (≈100 nm), enabling semi-transparent, flexible or tandem device construction.^10^ Moreover, over the past few years, the power conversion efficiency of Sb_2_Se_3_ and Sb_2_S_3_ solar cells was improved to 10.12%^11^ and 8%,^12^ respectively, and they are presently among the most rapidly developing PV technologies.

Perovskite solar cells (PSCs) is another technology currently being widely investigated and developed with future possibility to complement or even replace silicon-based devices.^13^ Compared to silicon, perovskite benefits from simple fabrication of various perovskite compositions, favourable optoelectronic properties, fast charge separation, slow recombination rate, and high tolerance of crystal defects in the layer.^14^ Hence, it is not surprising that perovskite solar cells exhibited one of the most significant progress in efficiency, from 3.8% in 2009 to 26.1% in 2023.^15^

In addition to the development of new and prospective light absorbers, recently, significant progress has been made in the field of hole-transporting materials (HTMs) for solar cells due to the novel concept of the hole-transporting monolayer. The carbazole molecule, containing a phosphonic acid anchoring group, can attach to various metal oxide surfaces and form a one-molecule-thick layer.^16^ This innovation had a significant impact on the improvement of p-i-n perovskite solar cells and at the same time further accelerated development of self-assembled (SAM) hole-transporting materials.^17^ However, the efficiency of the solar cells is also strongly dependent on the type of semiconductor selected as electron-transporting material (ETM).^18^ Furthermore, the choice of ETMs is significantly more limited compared with hole-transporting analogues. Among investigated organic electron transport molecules, three of the biggest groups are fullerene derivatives as well as naphthalene and perylene diimides.^19^ Fullerene-based acceptors have limited options for the manipulation of molecular structure, often poor morphological stability, and high synthesis cost.^19^ Thus, various strategies of designing small naphthalene and perylene diimide molecules were developed; however, insufficient photovoltaic properties have hindered their broader application.^20^

Fluorene is quite a well-known aromatic molecule with a delocalized π-conjugated system, which can take part in transporting charge carriers in solar cells.^21^ Over the past two decades, fluorene-based derivatives were explored mostly as small hole-transporting molecules and as moieties for polymeric acceptors for application in photovoltaics.^22,23^

Another interesting candidate in the search for the most suitable central core for small-molecule electron-transporting materials is fluorenone. Fluorenones possess several similar advantages as fluorenes, including ease of structural modification, high thermal stability and good charge transport and optoelectronic properties. They are extensively used as the central core for functional materials in organic field-effect transistors, light-emitting diodes, and biological sensors.^24^ For example, Chen *et al.* published imide-functionalized fluorenone and its cyanated derivative, designed as electron-accepting building blocks for n-type organic semiconductors.^25^ Both materials featured a deep-lying lowest unoccupied molecular orbital energy level at −3.68 eV and −4.05 eV, respectively. Additionally, fluorenone has been tested as a chromophore in A–D–A–D–A molecules applied in dye-sensitized and organic solar cells.^26^ Overall, these results indicate that the fluorenone fragment is a promising acceptor unit for the construction of high-performance n-type semiconductors.

To date, SAM materials were introduced as very effective hole-transporting materials, and carbazole-based SAMs such as [2-(9*H*-carbazol-9-yl)ethyl]phosphonic acid (2PACz) or [4-(3,6-dimethyl-9*H*-carbazol-9-yl)butyl]phosphonic acid (Me-4PACz) are currently very widely used in inverted perovskite and perovskite/Si tandem solar cells.^16,27^ Consequently, the success of HTM SAMs was an important factor that prompted us to put more effort into the investigation of new n-type semiconductors forming SAMs. In the current publication, we explore n-type SAMs based on fluorene or fluorenone chromophores and containing phosphonic acid anchoring groups. Their energetics, thermal and monolayer forming properties as well as potential for applicability as n-type semiconductors in different emerging photovoltaic technologies are investigated.

## Results and discussion

**Scheme 1:**
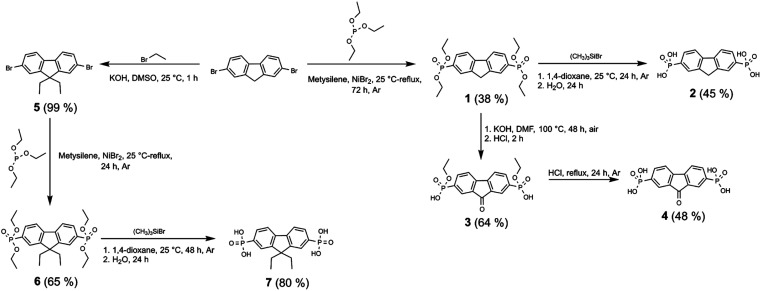
Synthetic route for the synthesis of fluorenes 2, 7 and fluorenone 4 containing phosphonic acid anchoring groups.

**Scheme 2:**
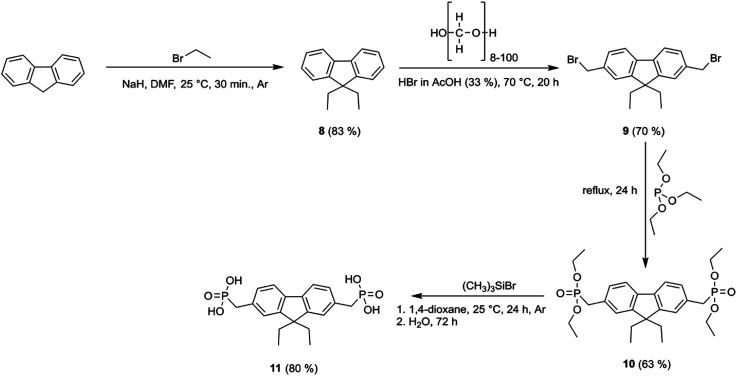
Synthetic route to fluorene-based derivative 11.

Synthesis of fluorenone- and fluorene-based compounds 2, 4, 7, and 11 was carried out according to the procedure described as follows (Schemes 1 and 2). Fluorene semiconductor 2 was synthesized by a two-step route, starting with the phosphonylation of 2,7-dibromofluorene with triethyl phosphite using NiBr_2_ to give intermediate 1. The hydrolysis of ethyl ester groups using an excess of bromotrimethylsilane in 1,4-dioxane, followed by workup in water, produced fluorene-2,7-diphosphonic acid (2).

Under alkaline conditions, the intermediate 1 was oxidized to a fluorenone derivative 3. Fluorene diphosphonic acid (4) was obtained by conducting further hydrolysis of the formed intermediate *via* reflux in HCl.

To prepare diethyl-fluorene with phosphonic acid anchoring groups at 2,7 positions (Scheme 1), dibromofluorene was alkylated using KOH and bromoethane in anhydrous DMSO at ambient conditions. Afterwards, intermediate 5 was phosphorylated *via* Michaelis–Arbuzov reaction, and phosphonium 6 was isolated. Next, fluorene dialkylphosphonate was transformed into the target product 7 by McKenna reaction. Lastly, fluorene 11 with unconjugated phosphonic groups was obtained in four steps from fluorene (Scheme 2), which was alkylated in DMSO using sodium hydride, and intermediate 8 was isolated. Bromomethyl group was introduced into intermediate 8 using paraformaldehyde and HBr in acetic acid, affording fluorene derivative 9. Finally, phosphonate 10 was formed by Michaelis–Arbuzov reaction, which was then converted to the appropriate diphosphonic acid 11 by McKenna reaction.

**Fig. 1:**
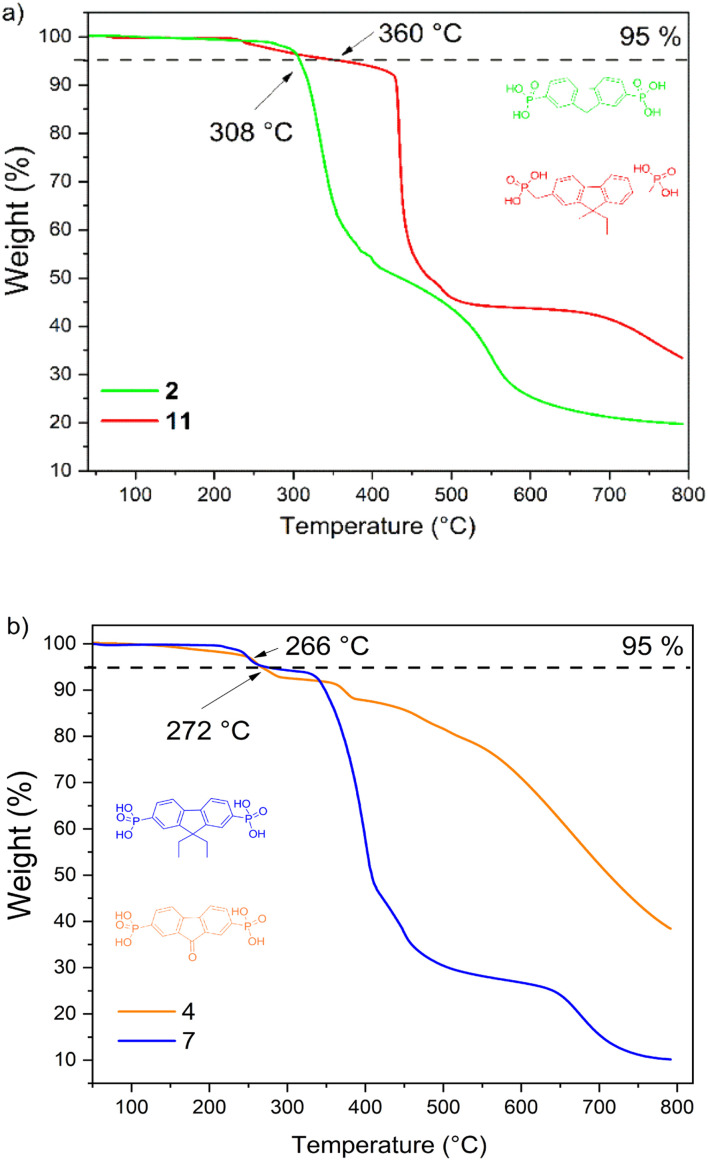
TGA curves of 2, 4, 7, and 11.

The thermal stability of the fluorine- and fluorenone-based derivatives was evaluated by thermogravimetric analysis (TGA) under nitrogen atmosphere. The weight loss curves are shown in Fig. 1(a and b), which reveal that semiconductors 2, 4, 7, and 11 demonstrate good thermal stability with decomposition temperatures ranging from 260 to 360 °C. TGA curves indicate that the materials degrade in several steps, and it is related to the elimination of substituents. The start of weight loss, which varies from 215 °C to 420 °C, can be assigned to the decomposition of phosphonic acid. From the TGA curves, mass losses calculated during the first decomposition step constitute ≈31 g mol^−1^ and are close to the molar mass of hydroxyl groups (34.01 g mol^−1^), indicating incipient decay of phosphonic acid from the compound structures of 4, 7, and 11. In the case of fluorene 2, the decomposition of phosphonic acid takes place in the range of 404–574 °C. Overall, these results suggest that new synthesized compounds are sufficiently thermally stable to meet stability requirements for application in PV devices.

We investigated the capability of semiconductors 2, 4, 7, and 11, containing the phosphonic acid anchoring groups, to bond to the surface of chalcogenide light absorbers or metal-oxide conductors and semiconductors. The surfaces of ITO, TiO_2_, Sb_2_Se_3_ and Sb_2_S_3_ were modified with these SAMs, and their water droplet contact angle results were compared with non-modified samples (Table 1).

**Table 1:** Contact angle measurements of water and perovskite precursor droplet on different surfaces

Water droplet					
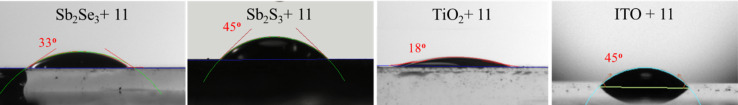					
Substrate	Control sample	2	4	7	11
ITO	31°	23°	25°	23°	45°
TiO_2_	Total wetting	9°	5°	3°	18°
Sb_2_Se_3_	58°	16°	13°	17°	33°
Sb_2_S_3_	24°	18°	26°	17°	45°
**Perovskite precursor droplet**					
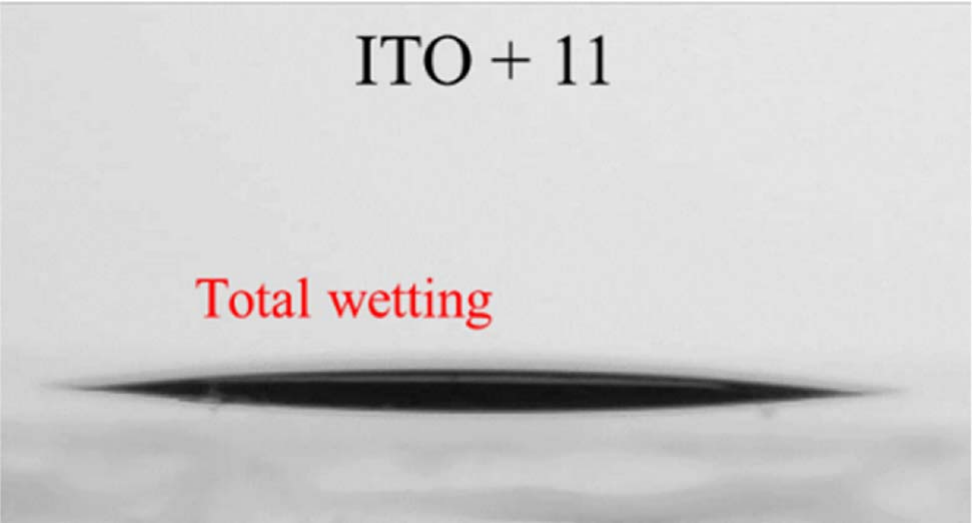					
ITO	Total wetting	Total wetting	Total wetting	Total wetting	Total wetting

The measured contact angle values are significantly lower for all Sb_2_Se_3_ surfaces modified by SAMs compared with the pristine Sb_2_Se_3_ sample, indicating the improved substrate wettability compared with pristine Sb_2_Se_3_ surface. In the case of ITO and Sb_2_S_3_ samples, the contact angle values decreased after monolayers from derivatives 2, 4, and 7 were formed. When fluorene 11 was used, ITO and Sb_2_S_3_ substrate film surfaces became slightly more hydrophobic (45°) in both cases. Moreover, compared to the contact angle results with other investigated derivatives, the monolayer formed by fluorene 11 was most hydrophobic.

To get a better idea for why there is a significant difference in the contact angle results between fluorene 11 and the other tested materials, molecular geometry calculations were done using density functional theory (DFT).

**Fig. 2:**
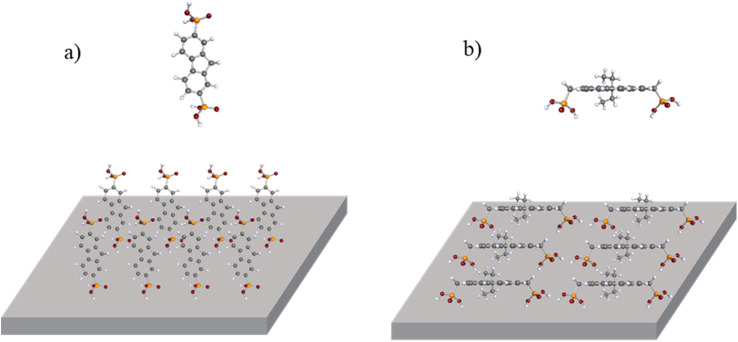
Molecular geometries of (a) ETM 2 and (b) ETM 11 with their schematic face-on molecular orientation on the ITO surface (calculated with Becke's three-parameter functional, B3LYP, and def2-SVP basis set in vacuum).

Data acquired from calculations suggest that semiconductor 11 can attach to the ITO surface using phosphonic acid anchoring groups simultaneously. This is due to methylene bridges that connect the phosphonic acid group to the conjugated part of the molecule. As a result, molecules tend to lay on their side, exposing the more hydrophobic part of the molecule. In contrast, all other molecules (2, 4, and 7) have anchoring groups attached directly to phenyl rings, limiting their surface-binding ability to only one phosphonic acid fragment (Fig. 2a and b). This translates to materials 2, 4, and 7 having lower contact angles, since unattached phosphonic acid is hydrophilic and facing upwards.

We also investigated the possibility to use these SAMs for interfacial modification between TiO_2_ and the light absorber, which can be beneficial for improved charge separation and reduced trap states of TiO_2_.^28^ After treatment with compounds 2, 4, 7, and 11, the TiO_2_ surface had slightly higher water droplet contact angles of 9°, 18°, 3°, and 5°, respectively, compared to the untreated TiO_2_ sample (Table 1), indicating the formation of monolayer on the surface.

Overall, it is evident that fluorene and fluorenone SAMs containing phosphonic acid functional groups can bind to investigated ITO, TiO_2_, Sb_2_Se_3_ and Sb_2_S_3_ surfaces.

Additionally, to obtain more data about the suitability of the SAM-coated ITO substrate for perovskite deposition, we measured the contact angles of non-modified and modified ITO with SAMs 2, 4, 7, and 11 using perovskite precursor solution instead of water. With this measurement, we aimed to confirm that the investigated materials, deposited on top of ITO, will not significantly increase the hydrophobicity of the surface, thus making it difficult to deposit uniform perovskite film on top. Poor surface wetting can result in worse layer quality, enabling large-scale pinholes in perovskite film and, consequently, an overall drop in PSC device performance.^29^ All modified ITO samples demonstrated total wetting, which is favourable for uniform perovskite layer deposition. The retained good wettability of the ITO surface after its modification is mainly due to the presence of phosphonic acid groups, which facilitated distribution of the perovskite solution.

**Fig. 3:**
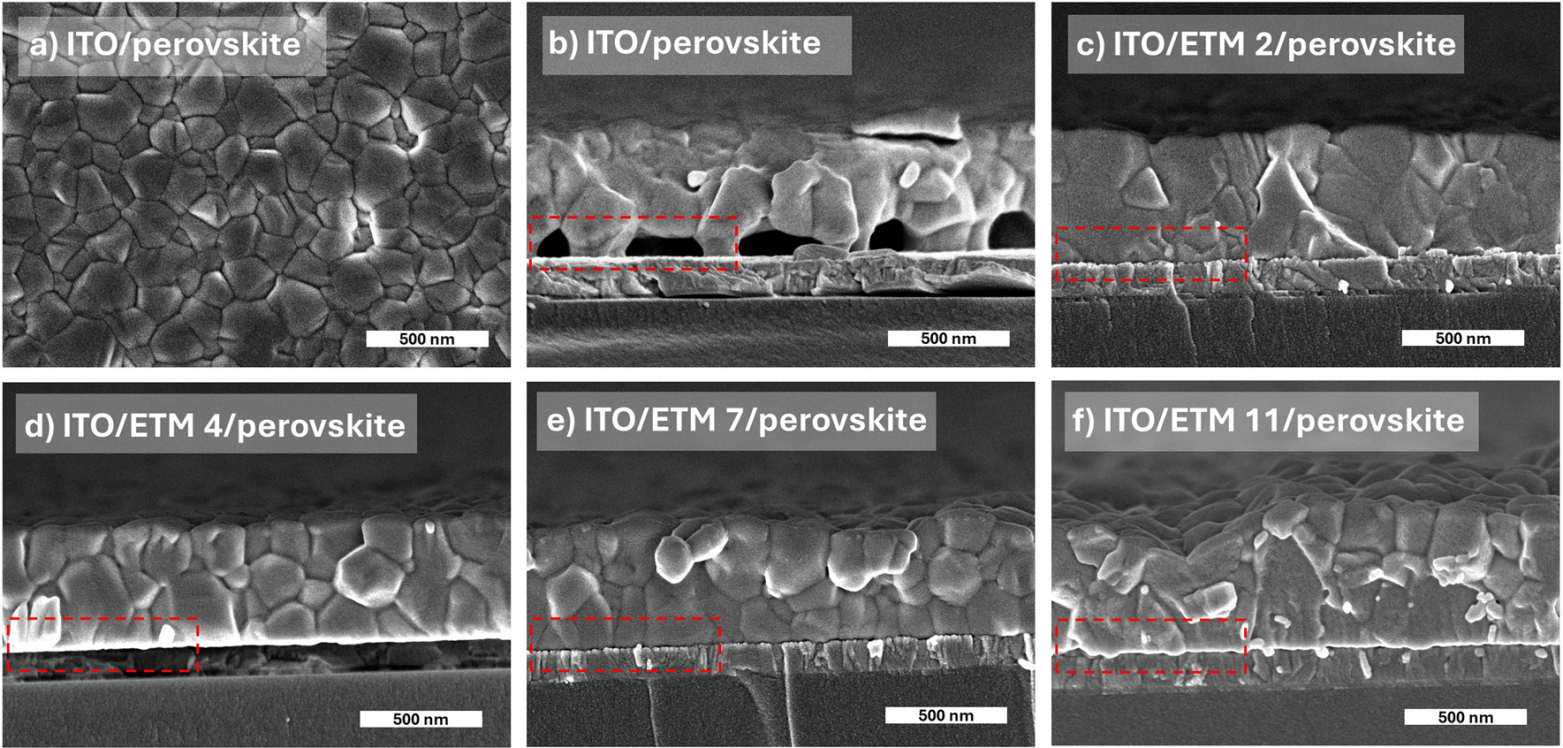
(a) Top-view SEM image of ITO/perovskite; (b) cross-sectional SEM images of ITO/perovskite and (c–f) ITO/ETMs/perovskite.

To examine the influence of semiconductors on the passivation effect for perovskite film morphology, scanning electron microscopy (SEM) was performed, and the obtained results are shown in Fig. 3. Due to the poor ITO and perovskite interaction, the layer is not closely packed and results in large pinholes at the interface (Fig. 3b). In contrast, the free phosphonic acid of the compounds interact with the absorber layer, resulting in less voids formed at the ITO/ETMs/perovskite interface and improved junctions (Fig. 3c–f), indicating that the compounds containing phosphonic acid interact with perovskite during the crystallization process and impact the perovskite morphology.

We employed transient photoluminescence (TRPL) spectroscopy to assess whether ETM monolayers solely serve as carrier extraction layers or passivate perovskite, thereby enhancing its quality. The obtained ITO/ETM-SAM/perovskite results were compared with glass/perovskite and ITO/perovskite samples. Excitation of the sample was performed from the interface side. The control sample of a neat perovskite film on glass reached a TRPL lifetime of more than 1 μs, which is associated with the Shockley–Read–Hall (SRH) recombination.^30^ In agreement with previous reports, the considerably long lifetime obtained indicates low recombination losses at glass interfaces, suggesting a high quality of the perovskite layer.^31^ In contrast, the lifetime of perovskite deposited on ITO is much shorter, decaying in approximately 130 ns. The pronounced photoluminescence quenching can be attributed to poor quality of the ITO/perovskite interface, as evidenced by SEM images (Fig. 3b). This poor quality could be manifested by the presence of empty spaces and imperfect coverage between the perovskite and ITO substrate, indicating a weak interaction between both surfaces. Such a poor perovskite coverage can result in the formation of dangling bonds and defects at the interface, thereby significantly impeding carrier transport across the layers and consequently leading to increased losses attributed to non-radiative recombination. We suggest that the observed reduction in TRPL decay mainly arises from interface-related phenomena rather than bulk properties. This is supported by the formation of a dense film with very few imperfections within the bulk perovskite structure, as illustrated in the SEM image provided (Fig. 3c–f).

**Fig. 4:**
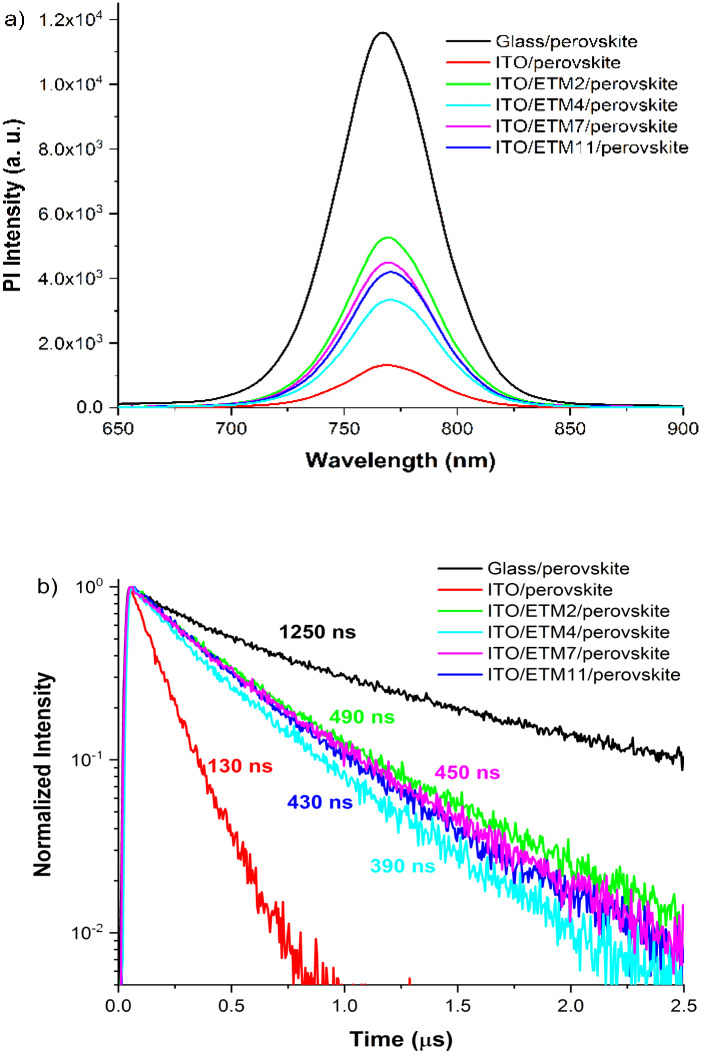
Comparison of ITO/ETM-SAMs/perovskite and glass/perovskite: (a) steady-state photoluminescence (PL) spectra and (b) TRPL kinetics of samples.

When the monolayers of ETM 2, 4, 7 or 11 are introduced between the ITO and perovskite interface, the average PL lifetime is substantially extended to 490 ns, 390 ns, 450 ns and 430 ns, respectively, as illustrated in Fig. 4b, compared to the ITO/perovskite sample. Notably, all samples demonstrate comparable monoexponential decays, regardless of the structural variation in the interlayer SAM. This is a rather unexpected result, as typically observed PL transients for perovskite interfaced with charge selective contacts display significantly varying slopes, indicative of a combination of nonradiative and radiative decays, as well as carrier extraction process.^31,32^ Hence, the absence of evident TRPL decay variation over the measured timescale indicates a single dominating process, most likely attributable to SHR recombination.^33^ In the case of hole extraction process with SAM, one can expect acceleration of the initial TRPL decay component, compared to perovskite interfaced with glass or ITO samples.^27,34^ Therefore, the prolonged monoexponential PL lifetime observed compared to the ITO/perovskite interface suggests that incorporated SAMs suppress non-radiative recombination by reducing perovskite surface defects, thus functioning as an interface-passivating agent rather than just a carrier-extracting layer.^16^ This observation is further supported by SEM cross-section images, which reveal a significantly improved perovskite adhesion to the ITO/SAM surface, potentially reducing surface defects and lowering the nonradiative recombination channel. Given that our SAMs are designed for facilitating electron transport and considering previous findings on perovskites incorporating oxide electron-transport layers,^35^ it is possible to expect that carrier transfer through the SAM may occur significantly faster than the temporal resolution attainable in our current experiment. However, asserting that perovskite passivation occurs within the observable several-hundred-nanosecond timeframe may not be entirely accurate, because other processes such as accumulation of charge carriers at interfaces might occur, which hinder electron transfer from perovskite to the selective contact. This accumulation could potentially lead to interfacial recombination, thereby complicating the differentiation between these two processes as well. Hence, further understanding about electron transfer through SAM necessitates a more thorough analysis of carrier transport involving diverse SAM compositions.

To study the energy level distribution, the redox potentials (summarized in ESI†) of fluorene and fluorenone derivatives were measured by cyclic voltammetry, and LUMO/HOMO energy levels were estimated. These values do not represent any absolute solid-state electron affinity but can be used to compare different compounds in relation to one another.

**Fig. 5:**
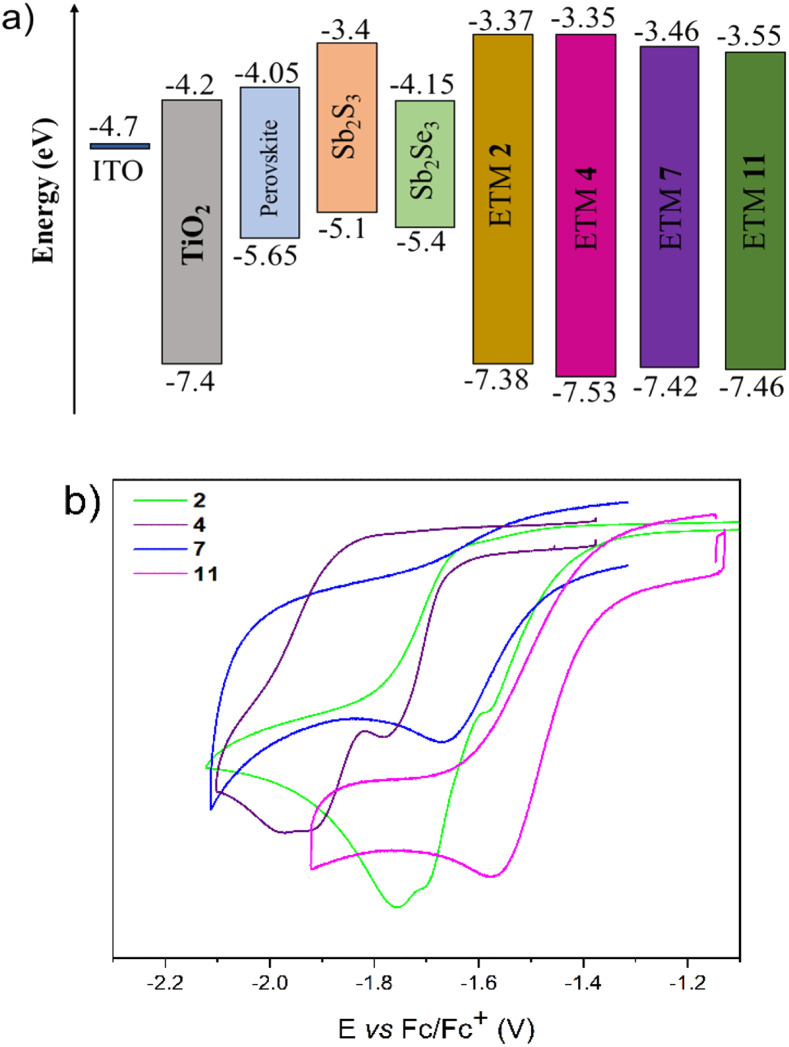
(a) Energy band diagrams of ETMs 2, 4, 7, and 11 and absorption layers and (b) redox voltamperograms of those investigated materials.

As shown in Fig. 5b, all compounds exhibit irreversible reduction waves. Accordingly, the LUMO energies of the designed ETM 2, 4, 7, and 11 were −3.37 eV, −3.35 eV, −3.46 eV, and −3.55 eV, while HOMO values were calculated to be −7.38 eV, −7.53 eV, −7.42 eV, and −7.46 eV, respectively (Fig. 5a). Consequently, the *E*_LUMO_ values of the semiconductors synthesized in this research are close to those for Sb_2_S_3_, revealing the favourable energetics for efficient electron transfer from the absorption layer to cathode. Semiconductors 2, 4, 7, and 11 can also efficiently mediate electron transport between perovskite or Sb_2_Se_3_ and electrode layers. The HOMO values of the synthesized molecules are over −7 eV, implying that these molecules can effectively block hole injection from getting into the transporting layer. Utomo *et al.* reported an n-type anthraquinone-based semiconductor (PAAQ) with similar LUMO (−3.52 eV) and HOMO (−6.79 eV) energy values. This semiconductor has achieved a promising efficiency (16%) in perovskite solar cells even with sufficiently low energy level values,^36^ suggesting the suitability of semiconductors 2, 4, 7, and 11 for application in PSCs as well.

**Fig. 6:**
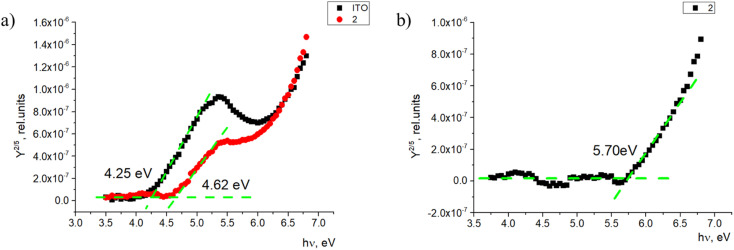
Photoemission yield spectra of (a) the ITO-covered glass and monolayer sample and (b) the photoemission yield spectrum of 2.

Additionally, energy level value measurements were done for the monolayer and thin film samples. The ionization energy level measurements were carried out in a self-made photoemission yield spectroscopy (PYS) and intrinsic photoconductivity measurement system. Detailed information about these measurements can be found elsewhere^37^ and for the sample preparation in ESI.† Monolayer samples on ITO covered glass substrates were prepared, and the photoemission yield spectral dependence was obtained for each sample. Two things can be observed in the spectrum. One, the photoemission spectrum shifts when ITO is covered with the investigated compounds (Fig. 6a). In this case, the ITO work function increases by 0.37 eV. This could be related to the material-created dipole moment at the sample surface, which interferes with photoemission, and thus, a higher photon energy is required to extract electrons from ITO.

Second, when ITO is covered with the investigated material, there is a second threshold where the photoemission yield sharply increases. This signal comes from the monolayer itself. As the photoemission spectrum here consists of two spectra—one created by ITO and the second created by the compound, it is possible to subtract the ITO photoemission to get the photoemission spectrum of the material itself (Fig. 6b). From this, the ionization energy level value of the monolayer can be obtained. In this case, the ionization energy level value of 2 in the monolayer sample is 5.70 eV. For fluorenes 2, 4, and 7, the ionization energy is similar, within 0.1 eV (between 5.60 and 5.70 eV; Fig. S1 and S2†). The only difference is the ionization energy for fluorenone 11, which was 5.99 eV. As shown in Fig. 2, the way fluorenone 11 attaches to the surface of ITO is different from other compounds. This could be the main reason for the ionization energy level difference.

**Fig. 7:**
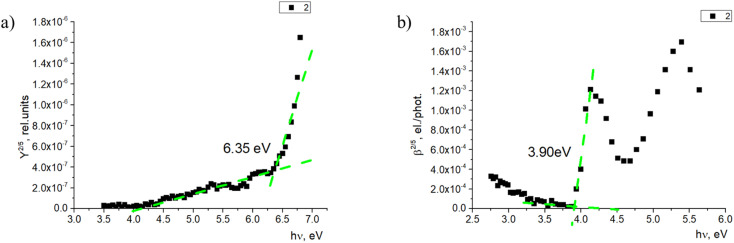
(a) Photoemission yield spectrum and (b) photoconductivity spectrum of 2.

The photoemission yield spectra were measured for thin-film samples. In this case, the films were thick enough (400–500 nm) to observe photoemission from the materials alone, without the signal coming from ITO (Fig. 7a). Here, the ionization energy level values of fluorenes 2, 4, and 7 again were similar and within 0.1 eV—in this case, between 6.35 and 6.45 eV. The ionization energy level of fluorenone 11 is 0.4 eV lower, at 5.96 eV. This could be related to material 11 having an additional methylene group between 9,9-diethyl-9*H*-fluorene and the phosphonic acid group. The difference in side chains could lead to the difference in the film formation and molecule interaction, which leads to the noticeable decrease in ionization energy level value.

The electron affinity energy value for the materials in thin films was obtained indirectly from the values of the ionization energy and energy gap. It has been shown^38,39^ that photoconductivity measurements allow estimating the energy gap, as the photoconductivity quantum efficiency *β* is proportional to the exciton dissociation probability near the threshold energy. The measurements and data processing have been described in detail.^37^ The obtained results are presented in Table 2. For all the studied materials, the energy gap is above 3.50 eV. Such high bandgap values allow avoiding issues with parasitic absorption in the charge carrier layer when materials like these are used.

**Table 2:** Energy level values of 2, 4, 7, and 11 in thin films

Material	Ionization energy level (monolayer), eV ± 0.03 eV	Ionization energy level (film), eV ± 0.03 eV	Electron affinity (film), eV ± 0.05 eV	Energy gap, eV ± 0.03 eV
2	5.70	6.35	2.45	3.90
4	5.61	6.44	2.72	3.72
7	5.65	6.34	2.51	3.83
11	5.99	5.96	2.33	3.63

**Fig. 8:**
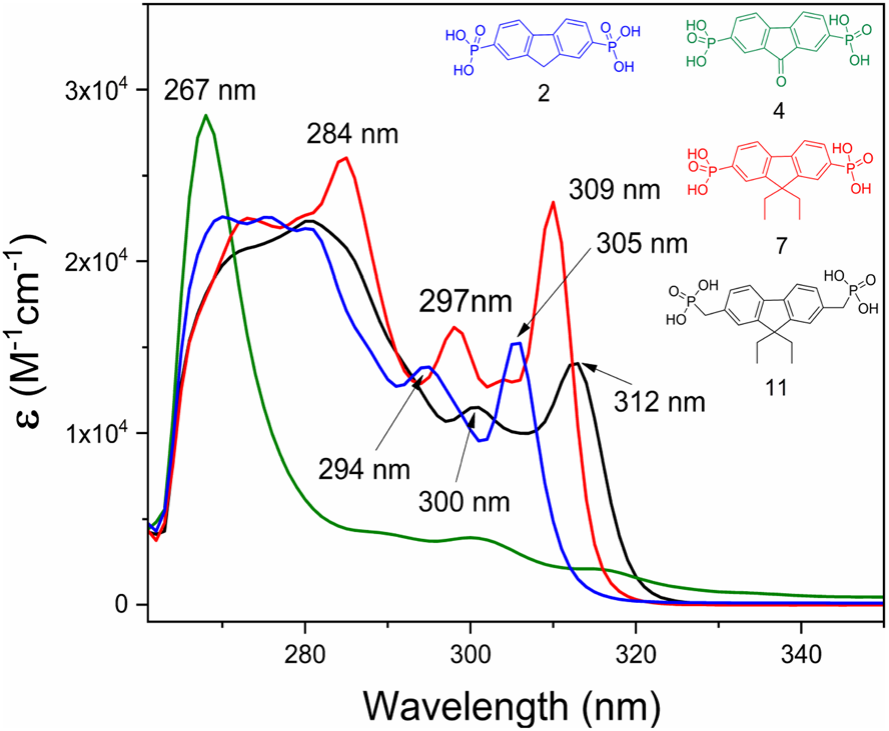
UV-Vis spectra of compounds 2, 4, 7, and 11 in DMF solution (*M* = 10^−4^).

Fluorenone 4 exhibits a relatively intense narrow absorption peak at 267 nm, assigned to the ε–ε* transitions of the central fragment (Fig. 8). The absorption spectra of fluorene derivatives showed three absorption maxima, of which the two at 294–312 nm interval are moderately intense, followed by a stronger and wider absorption at the shorter wavelength region. These absorptions are attributed to the π–π* transition associated with the fluorenyl fragment. Interestingly, a small hypsochromic shift for compounds 2 and 7 was observed, compared to fluorene 11, which contains the CH_2_ spacer. These shifts can be related to weaker influence of electron-accepting phosphonic acid anchoring groups.

## Experimental section

All information is provided in the ESI file.†

## Conclusions

To conclude, fluorene and fluorenone derivatives containing phosphonic acid anchoring groups were synthesized and investigated for their suitability to be used as electron transporting SAM materials with different light absorbers. The investigated organic materials demonstrate sufficient thermal stability and suitable electrochemical properties for effective electron transport from perovskite or chalcogenide absorber layers to the electrode. Water contact angle measurements indicate that the investigated materials can be successfully deposited on metal oxides (ITO and TiO_2_) as well as antimony chalcogenides (Sb_2_S_3_ and Sb_2_Se_3_). Transient photoluminescence analysis indicates that perovskite film morphology improved due to the passivating effect of ETM monolayers; fluorene derivative 2 without alkyl groups demonstrated the best passivation efficiency among the investigated materials. In summary, fluorene and fluorenone derivatives based on SAMs are promising materials for application in perovskite and antimony chalcogenide-based solar cells.

## Author contributions

Lauryna Monika Svirskaite: synthesis, investigation, visualization, data processing, writing – original draft. Ernestas Kasparavičius: investigation, visualization, writing. Matas Steponaitis: modeling, writing. Raitis Grzibovskis: investigation, visualization, writing. Marius Franckevičius: investigation, writing. Arnas Naujokaitis: investigation. Atanas Katerski: investigation, resources. Smagul Karazhabov: resources, funding acquisition. Sajeesh Vadakkedath Gopi: investigation. Arturs Aizstrauts: data processing. Aivars Vembris: resources. Vytautas Getautis: resources, funding acquisition. Tadas Malinauskas: supervision, conceptualization, writing – review & editing.

## Conflicts of interest

There are no conflicts to declare.

## Supplementary Material

RA-014-D4RA00964A-s001
