# Supplementary material for: Fluorene- and fluorenone-based molecules as electron-transporting SAMs for photovoltaic devices
Source: RSC Adv. 2024 May 10;14(21):14973–81. doi: 10.1039/d4ra00964a (PMC11082726; doi:10.1039/d4ra00964a)
Supplement: RA-014-D4RA00964A-s001 [file RA-014-D4RA00964A-s001.pdf]

Electronic Supplementary Material (ESI) for RSC Advances.
This journal is © The Royal Society of Chemistry 2024

Supporting Information

Fluorene and fluorenone-based molecules as electron transporting SAMs for photovoltaic devices

*Lauryna Monika Svirskaite[a], Ernestas Kasparavičius[a], Matas Steponaitis[a], Raitis Grzibovskis[d], Marius Franckevičius[e], Atanas Katerski[b], Arnas Naujokaitis[e], Smagul Karazhanov[c], Sajeesh Vadakkedath Gopi[b], Arturs Aizstrauts[d], Aivars Vembris[d], Vytautas Getautis[a], Tadas Malinauskas[a]**

[a]Department of Organic Chemistry, Kaunas University of Technology, Radvilenu pl. 19, Kaunas, 50254, Lithuania.

[b]Department of Material and Environmental Technology, Tallinn University of Technology, Ehitajate tee 5, Tallinn, 19086, Estonia.

[c]Department for Solar Energy, Institute for Energy Technology, PO BOX 40, 2027, Kjeller, Norway

[d]Institute of Solid State Physics, University of Latvia, Kengaraga st. 8, Riga, LV-1063, Latvia.

[e] Center for Physical Sciences and Technology, Saulėtekio Ave. 3, 10257, Vilnius, Lithuania

Materials and general procedures

Chemicals were purchased from Sigma-Aldrich and TCI Europe used as received without further purification. The course of the reactions products were monitored by TLC on ALUGRAM SIL G/UV254 plates and developed with UV light. Silica gel (grade 9385, 230-400 mesh, 60 Å, Aldrich) was used for column chromatography. The ^{1}H and ^{13}C NMR spectra were taken on Bruker Avance III (400 MHz) spectrometer at RT and all the data are given as chemical shifts in δ (ppm).

Elemental analysis was performed with an Exeter Analytical CE-440 elemental analyzer, model 440 C/H/N/. Electrothermal MEL-TEMP capillary melting point apparatus was used for determination of melting points. Thermogravimetric analysis (TGA) was performed on a Q50 thermogravimetric analyzer (TA Instruments) at a scan rate of 10 K min^{-1} in the nitrogen atmosphere. UV-Vis spectra were recorded on Shimadzu UV-2600 spectrophotometer. The samples of compounds were prepared from 10^{-4} M anhydrous DMF and microcells of 1 cm width were used for measurements.

Contact angles of water droplets on TiO_2, Sb_2S_3, or Sb_2Se_3 substrates and substrates coated with fluorene and fluorenone derivatives monolayer were estimated using system DSA25B from Kruess GmbH and analysis software "Drop Shape Analysis" version 1.1, while the contact angle of water and perovskite precursor droplets on non-modified and modified ITO samples were measured by an optical goniometer (The Ossila). Measurement range 5-180°; Measurement accuracy ±1°.The geometry optimization DFT calculations of compounds **2**, **4**, **7**, **11** were performed using TURBOMOLE version 7.0 software [1], with Becke's three parameter functional, B3LYP [2, 3], and def2-SVP [4, 5] basis set in vacuum. The surface morphology of thin films were carried out in a dual-beam system of a scanning electron microscope Helios Nanolab 650 equipped with an EDX spectrometer from Oxford Instruments. Was using 2keV voltage for ITO/perovskite and ITO/ETMs/perovskite samples. A few nanometers of chromium were sputtered on the cross-section due to the non-conductive/ low conductivity surface. Samples were placed in a chamber up to $2E^{-5}$ mbar pressure.

Cyclic voltammetry measurement

Cyclic voltammetry (CV) measurements were carried out by a three-electrode assembly cell from Bio-Logic SP-150. The measurements were carried out at a glassy carbon electrode in DMF solutions containing 0.1 M tetrabutylammonium hexafluorophosphate as an electrolyte, Pt as the counter electrode and as the reference electrode. Each measurement was calibrated with ferrocene (Fc) and potentials calculated to Fc^+/Fc. Conversion factors: ferrocene in DMF *vs* SCE 0.45, SCE *vs* SHE: 0.244, SHE *vs* vacuum: 4.43 [6, 7]. The half-wave potential $E_{1/2\ vs\ Fc}$ was obtained as an average value between maxima of cathodic and anodic potentials: $E_{1/2\ vs\ Fc} = 1/2(E_{red} - E_{ox})$. The optical band gaps $E_{g\ opt.} = \frac{1240}{\lambda}$ was estimated from the edges of electronic absorption spectra in DMF solution. E_{LUMO} was calculated from the equation $E_{LUMO} = (E\ vs\ Fc/Fc^+ + (solvent\ vs\ SCE) + 0.244 + 4.43$. E_{HOMO} calculated from the equation $E_{LUMO} = E_{LUMO} - E_{g\ opt.}$.

Sample preparation for energy level measurements

For the ionization energy level measurements, the samples were made on indium tin oxide (ITO) covered glass (Präzisions Glas and Optik GmbH, 20 Ω/sq.). Samples for photoconductivity measurements were made on slide glasses with the Al electrodes deposited on them. The Al electrodes were deposited using thermal evaporation in the vacuum method and their thickness was 100 nm. The distance between Al electrodes was 2mm. The studied materials were dissolved in methanol creating a solution with a concentration of 1 mg/ml. The films were formed by the drop-casting method, keeping the glass substrate on a hotplate at 80°C temperature. The film preparation was done in Ar glovebox. The thickness of the samples was in the range between 400 nm and 500 nm. The monolayer samples were made by dissolving the studied materials in methanol with the concentration of 0.5 mg/ml. The layer was formed by spin-coating method. The rotation speed was 2000 rpm, the acceleration was 2000 rpm/s, and the rotation time was 60s. The samples were dried on a hotplate at 80°C temperature for 15 minutes. Afterwards the excess material was rinsed off using methanol.

Energy level measurements of ETMs

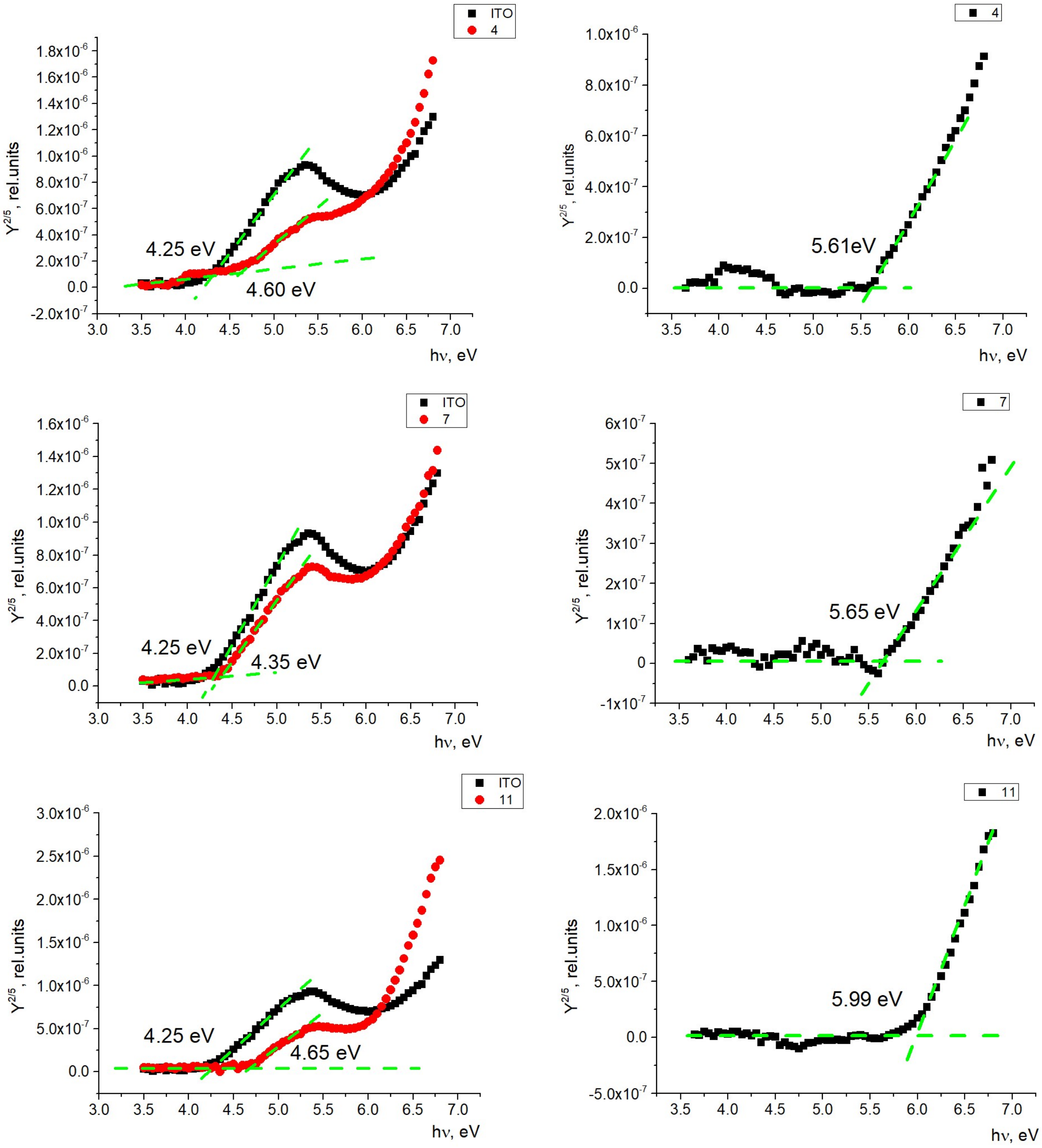

Figure S1 Photoemission yield spectra of the ITO covered glass and monolayer samples (left side), and photoemission yield spectrum of ETMs (right side)

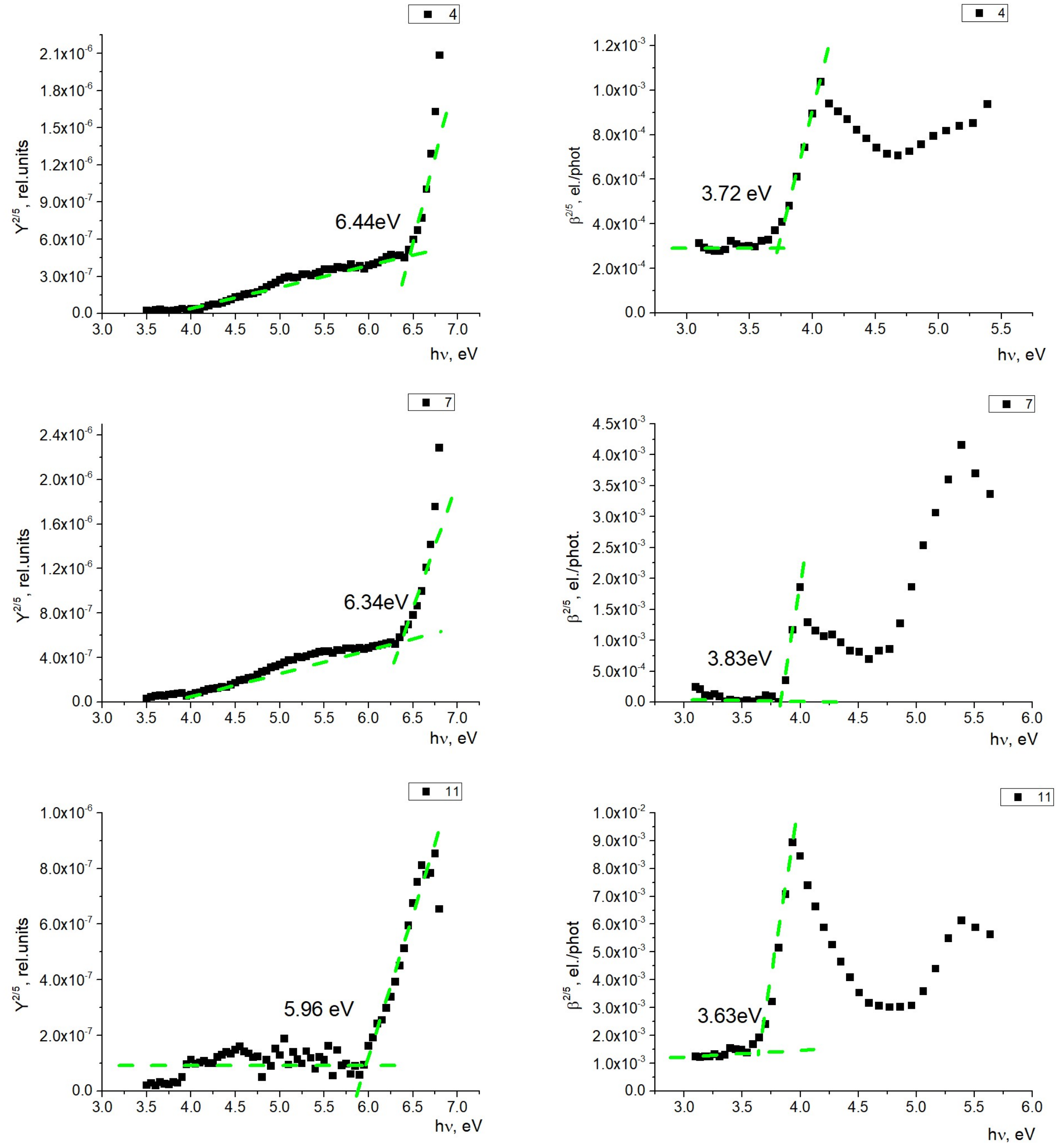

Figure S2 Photoemission yield spectra of ETM thin films (left side), and photoconductivity spectra of ETM thin films (right side)

Time-resolved photoluminescence (TRPL) measurement

To investigate the fluorescence decay kinetics on the microsecond time scale, the photoluminescence dynamics were measured using a streak camera system (Hamamatsu C5680) with single-sweep (M5677) unit coupled to a spectrometer. A femtosecond Yb:KGW oscillator (Light Conversion Ltd., Vilnius, Lithuania) generating 80 fs

duration pulses at 1030 nm, which were frequency doubled to 515 nm (HIRO harmonics generator, Light Conversion Ltd.), was used for the sample excitation. The laser pulses were attenuated and focused into an ~100 μm spot on the sample, resulting in an average excitation power of approximately 10 nJ/cm^2. The streak image processing was applied using HPDTA 9.3 software (Hamamatsu Photonics, K.K.) to obtain time-resolved fluorescence spectra. The temporal resolutions of the streak images within the 2 us time range was around ~15 ns.

Preparation of Sb_2S_3 and Sb_2Se_3 thin films

ITO covered (sheet resistance=20 Ω sq^{-1}) soda-lime glass substrates were cleaned with deionized water, ethanol, deionized water, H_2SO_4 (1 %), and rinsed with deionized water before drying at 105 °C in air. Then, a dense, compact TiO_2 layer was grown on glass/ITO by ultrasonic spray pyrolysis in air from 0.2 M titanium tetraisopropoxide (98 %) precursor and 0.2 M acetylacetone (99 %) dissolved in ethanol (96.6%). After deposition, the glass/ITO/TiO_2 stack was annealed at 450 °C for 30 min in air to assure the formation of the anatase phase. Amorphous layers of Sb_2S_3 were deposited by ultrasonic spray pyrolysis in air from a solution of $SbCl_3$ (99,999 % ThermoScientific) and $SC(NH_2)_2$ (99,99 %), Sb/S molar ratio 1:3, dissolved in methanol (99.8 %). The 1M stock solution from $SbCl_3$ in methanol was prepared inside a glovebox with controlled humidity (<7 ppm). The precursor solutions were prepared in room conditions. Ultrasonically nebulized precursor solution was transported to the preheated to 185 °C glass/ITO/TiO_2 substrates with helps of carrier gas (compressed air with flow rate of 2.5 L/min). The Sb_2S_3 thin films were crystallized by rapid annealing approach at 270 °C for 6 min in N_2 atmosphere. [8,9]. Sb_2Se_3 absorber layers were deposited from a Sb_2Se_3 powder (5 N, Sigma-Aldrich) using Vapour Transport Deposition (VTD) setup under vacuum level of 10^{-5} mbar. A 30 nm thick Sb_2Se_3 seed layer was first deposited at 280 °C substrate temperature and 400 °C source temperature. Next, Sb_2Se_3 film with ~1.1 μm thickness was deposited at 460 °C substrate temperature and 500 °C source temperature for 5 minutes.

Preparation of $Cs_{0.05}(MA_{0.17}FA_{0.83})_{0.95}Pb(I_{0.83}Br_{0.17})_3$ precursor solution

Perovskite precursor solution was prepared by using 0.507 g PbI_2, 0.074 g $PbBr_2$, 0.023 g MABr, 0.172 g FAI and 0.042 g CsI in 1 ml of anhydrous DMF:DMSO (4:1 volume ratio).

Preparation of ITO/ETM/perovskite substrates for Photoluminescence Spectroscopy and TRPL measurement

ITO glass substrates were cleaned for 15 minutes in an ultrasonic bath with 2 % Mucasol solution at ~ 40 °C. After rinsing with water, water and acetone, the substrates were further cleaned by annealing at 250 °C for 30 minutes and treated in an UV-ozone cleaner for 15 minutes. 60 µl of the ETM (0.5 mg/ml in methanol) solution was dropped uniformly on the ITO substrate and after 20 seconds, the sample was spin-coated at 2000 rpm for 30 seconds. Afterwards, the sample was annealed at 100 °C for 10 minutes, left for 1 minute to cool down, washed with 1 ml of pure methanol and substrate was dried under a stream of argon gas. Then, 60 µl of perovskite solution was spin-coated at 1000 rpm for 10 seconds, followed by 6000 rpm for 25 seconds. 10 seconds before the end of the spin-coating, 250 µl of chlorobenzene were dropped. The samples were annealed at 100 °C for 1 hour.

Preparation of Glass/perovskite substrate for Photoluminescence Spectroscopy and TRPL measurement

Corning© glass substrate was cleaned with 2 % Mucasol solution in water and after rinsing with water, water and acetone, the substrate was further cleaned by annealing at 500 °C for 30 minutes. Then, 80 µl of perovskite solution were spin-coated at 1000 rpm for 10 seconds, followed by 6000 rpm for 25 seconds. 10 seconds before the end of the spin-coating, 500 µl of chlorobenzene were dropped. The sample was annealed at 100 °C for 1 hour.

Preparation of ITO/ETM substrates for contact angle measurement

ITO glass substrates were cleaned for 15 minutes in an ultrasonic bath with 2 % Mucasol solution at ~ 40 °C. After rinsing with water, water and acetone, the substrates were further cleaned by annealing at 250 °C for 30 minutes and treated in an UV-ozone cleaner for 15 minutes. 60 µl of the ETM (0.5 mg/ml in methanol) solution was dropped uniformly on the ITO substrate and after 20 seconds, the sample was spin-coated at 2000 rpm for 30 seconds. Then, the samples were annealed at 100 °C for 10 minutes, left for 1 minute to cool down, washed with 1 ml of pure methanol and substrates were dried under a stream of argon gas.

The detailed synthetic procedures

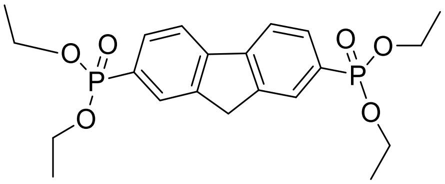

2,7-tetraethylphosphonofluorene (1)

2,7-Dibromofluorene (0.5 g, 1.54 mmol) was dissolved in mesitylene (5 ml) and triethyl phosphite (1.6 ml, 9.2 mmol) was added dropwise. The reaction mixture was purged with argon while $NiBr_2$ (0.024 g, 0.19 mmol) was added into the flask. Afterwards, the solution was refluxed for 72 hours under argon atmosphere and after reaction completion (TLC: acetone:*n*-hexane, 4:1), solvents were removed under reduced pressure. The crude product was dissolved in THF (30 ml) and filtered through celite. Material was purified by column chromatography (acetone:*n*-hexane, 4:1) yielding product as a white crystals (0.229 g, 38 %); m.p. 132-133 °C.
^{1}H NMR (400 MHz, DMSO-d_6) δ: 8.16 (d, J = 7.0 Hz, 2H), 7.97 (d, J = 13.0 Hz, 2H), 7.78 (dd, J = 12.6, 7.9 Hz, 2H), 4.12 (s, 2H), 4.10 – 3.98 (m, 8H), 1.25 (t, J = 7.0 Hz, 12H) ppm. ^{13}C NMR (101 MHz, DMSO-d_6) δ: 144.21, 144.05, 143.77, 143.75, 130.23, 130.12, 128.47, 128.28, 128.18, 126.62, 121.28, 121.12, 61.75, 61.70, 16.24, 16.18 ppm. Anal. calcd. for $C_{21}H_{28}O_6P_2$: C 57.53; H 6.44; found: C 57.41; H 6.36.

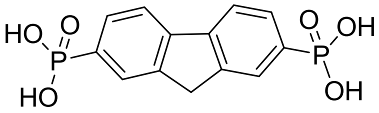

fluorene-2,7-diphosphonic acid (2)

2,7-Tetraethylphosphonofluorene (**1**) (0.2 g, 0.46 mmol) was dissolved in anhydrous 1,4-dioxane (3 ml) under argon atmosphere with followed by a dropwise addition of bromotrimethylsilane (0.6 ml, 3.9 mmol). The reaction mixture was stirred for 24 hours at 25 °C under argon atmosphere. After the termination of reaction (TLC: acetone:*n*-hexane, 4:1), distilled water was added dropwise until grey precipitate was formed and stirring continued overnight. Precipitate was filtered off and washed with distilled water to give 0.067 g (45 %) of light grey powder; m.p. 265-266 °C.

^{1}H NMR (400 MHz, DMSO-d_6) δ: 8.01 (d, *J* = 7.1 Hz, 2H), 7.89 (d, *J* = 12.6 Hz, 2H), 7.73 (dd, *J* = 11.5, 8.2 Hz, 2H), 7.12 (s, 4H), 4.01 (s, 2H) ppm. ^{13}C NMR (101 MHz, DMSO-d_6) δ: 143.47, 143.32, 142.72, 142.71, 133.81, 132.01, 129.48, 129.38, 127.46, 127.36, 120.41, 120.26, 36.44 ppm. Anal. calcd. for $C_{13}H_{12}O_6P_2$: C 47.87; H 3.71; found: C 47.75; H 3.66.

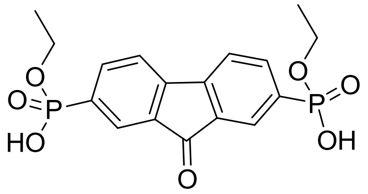

Diethyl (9-oxo-9*H*-fluorene-2,7-diyl)bis(hydrogen phosphonate) (3)

A mixture of 2,7-tetraethylphosphonofluorene (**1**) (0.5 g, 1.14 mmol) and KOH (0.765 g, 13,66 mmol) in DMSO (5 ml) was stirred at 100 °C under air atmosphere. After 48 hours (TLC: acetone:THF:methanol, 20:4:1), 3 ml of concentrated HCl was added of dropwise and it stirred for 2 hours. The solvent was removed under reduced pressure and product was poured into deionized water. The formed precipitate was filtered off and washed with deionized water to obtain product as orange solid (0.289 g, 64 %).

^{1}H NMR (400 MHz, DMSO-d_6) δ: 8.06 – 7.91 (m, 4H), 7.83 (d, *J* = 12.0 Hz, 2H), 3.94 (p, *J* = 7.2 Hz, 4H), 1.20 (t, *J* = 7.0 Hz, 6H) ppm. ^{13}C NMR (101 MHz, DMSO-d_6) δ: 191.54, 145.71, 138.35, 138.25, 134.45, 133.43, 133.28, 132.63, 125.98, 125.88, 122.17, 122.03, 61.06, 61.01, 16.32, 16.25 ppm. Anal. calcd. for $C_{17}H_{18}O_7P_2$: C 51.53; H 4.58; found: C 51.40 H 4.43.

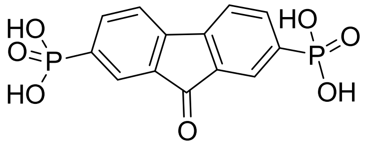

(9-oxo-9*H*-fluorene-2,7-diyl)diphosphonic acid (4)

Diethyl(9-oxo-9*H*-fluorene-2,7-diyl)bis(hydrogen phosphonate (**3**) (0.25 g, 0.63 mmol) and concentrated hydrochloric acid (1.3 ml) were refluxed overnight under argon atmosphere. Afterwards, solvent was partially evaporated, formed precipitate filtered off and washed with deionized water to obtain the product as yellow crystals (0.103 g, 48 %); m.p. >405 °C.

^{1}H NMR (400 MHz, DMSO-d_6) δ: 10.22 (s, 4H), 7.96 – 7.81 (m, 4H), 7.77 (d, *J* = 11.9 Hz, 2H) ppm. ^{13}C NMR (101 MHz, DMSO-d_6) δ: 191.90, 145.14, 145.12, 137.74, 137.63, 137.11,

135.31, 133.15, 133.01, 125.56, 125.45, 121.63, 121.49 ppm. Anal. calcd. for $C_{13}H_{10}O_7P_2$: C 45.90; H 2.96; found: C 45.83; H 2.86.

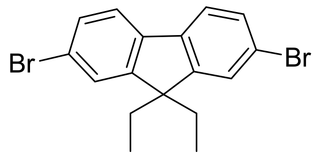

2,7-dibromo-9,9-diethyl-9*H*-fluorene (5)

2,7-Dibromofluorene (1 g, 3.08 mmol) was dissolved in anhydrous DMSO (10 ml) followed by addition of bromoethane (0.7 ml, 9.23 mmol) and ground KOH (0.518 g, 9.25 mmol). The reaction mixture was stirred at room temperature for 1 hour. After the termination of the reaction (TLC: *n*-hexane), reaction mixture was extracted with ethyl acetate. The organic layer was dried over anhydrous Na_2SO_4 and the solvent was evaporated under reduced pressure. The crude product was purified by column chromatography (*n*-hexane) resulting in yellow-white crystals (1.13 g, 99 % yield); m.p. 153-154 °C.

^{1}H NMR (400 MHz, DMSO-d$_6$) δ: 7.80 (d, J = 8.1 Hz, 2H), 7.68 (s, 2H), 7.54 (d, J = 8.1 Hz, 2H), 2.04 (q, J = 7.2 Hz, 4H), 0.18 (t, J = 7.2 Hz, 6H) ppm. ^{13}C NMR (101 MHz, DMSO-d$_6$) δ: 151.67, 139.20, 130.14, 126.11, 122.02, 121.01, 56.57, 31.36, 8.28 ppm. Anal. calcd for $C_{17}H_{16}Br_2$: C 53.72; H 4.24; found: C 53.66; H 4.17.

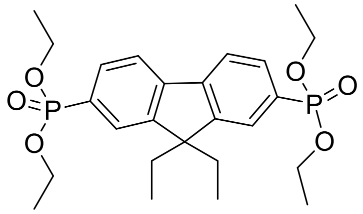

Tetraethyl(9,9-diethyl-9*H*-fluorene-2,7-diyl)bis(phosphonate) (6)

2,7-dibromo-9,9-diethyl-9*H*-fluorene (**5**) (1.1 g, 2.89 mmol) was dissolved in mesitylene (10 ml) and triethyl phosphite (1.5 ml, 8.67 mmol) was added dropwise. The reaction mixture was purged with argon while $NiBr_2$ (0.038 g, 0.17 mmol) was placed in to the flask. Afterwards, the solution was refluxed for 24 hours and after reaction completion (TLC: acetone:*n*-hexane, 1:1), mesitylene was removed under reduced pressure. The crude product was dissolved in acetone (15

ml) and filtered through celite. Afterwards material was purified by column chromatography (acetone:*n*-hexane, 1:1) yielding product as yellowish resin (0.941 g, 65 %).

^{1}H NMR (400 MHz, DMSO-d$_6$) δ: 8.09 (dd, *J* = 7.7, 3.2 Hz, 2H), 7.83 – 7.70 (m, 4H), 4.11 – 3.96 (m, 8H), 2.12 (q, *J* = 7.1 Hz, 4H), 1.23 (t, *J* = 7.0 Hz, 12H), 0.17 (t, *J* = 7.2 Hz, 6H) ppm.

^{13}C NMR (101 MHz, DMSO-d$_6$) δ: 150.21, 150.06, 143.65, 143.63, 130.77, 130.66, 129.05, 127.20, 125.88, 125.77, 121.17, 121.01, 61.76, 61.70, 56.31, 31.35, 16.17, 16.12, 8.24 ppm.

Anal. calcd. for $C_{25}H_{36}O_6P_2$: C 60.72; H 7.34; found: C 60.61; H 7.26.

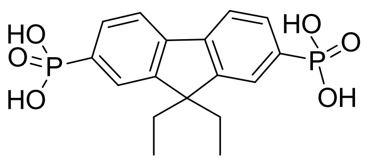

(9,9-diethyl-9*H*-fluorene-2,7-diyl)diphosphonic acid (7)

Tetraethyl(9,9-diethyl-9*H*-fluorene-2,7-diyl)bis(phosphonate) (**6**) (0.9 g, 1.82 mmol) was dissolved in anhydrous 1,4-dioxane (15 ml) followed by a dropwise addition of bromotrimethylsilane (6.2 ml, 36.4 mmol). The reaction mixture was stirred for 48 hours at 25 °C under argon atmosphere. After the termination of the reaction (TLC: acetone:n-hexane, 1:1), the solution were partially distilled under reduced pressure and deionized water was added dropwise until white precipitate was formed and stirring continued overnight. Precipitate was filtered off and washed with deionized water to give 0.603 g (80 %) of white crystals; m.p. 301-302 °C.

^{1}H NMR (400 MHz, DMSO-d$_6$) δ: 9.40 (s, 4H), 7.99 – 7.92 (m, 2H), 7.79 – 7.64 (m, 4H), 2.05 (dd, *J* = 14.0, 6.8 Hz, 4H), 0.22 (t, *J* = 7.2 Hz, 6H) ppm. ^{13}C NMR (101 MHz, DMSO-d$_6$) δ: 149.50, 149.36, 142.60, 142.58, 134.35, 132.55, 129.89, 129.78, 125.12, 125.01, 120.27, 120.12, 55.93, 31.62, 8.42 ppm. Anal. calcd. for $C_{17}H_{20}O_6P_2$: C 53.41; H 5.27; found: C 53.24; H 5.03.

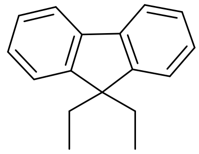

9,9-diethyl-9*H*-fluorene (8)

Under argon atmosphere, fluorene (1 g, 6.01 mmol) was dissolved in anhydrous DMF (10 ml), followed by addition of bromoethane (1.33 ml, 17.9 mmol). Afterwards, sodium hydride 60 %

dispersion in mineral oil (0.721 g, 30 mmol) was added in portions and the solution was stirred continuously for 30 minutes. After termination of the reaction (TLC: *n*-hexane), reaction mixture was extracted with ethyl acetate. The organic layer was dried over anhydrous Na_2SO_4 and the solvent was evaporated under reduced pressure. The crude product was purified by column chromatography (*n*-hexane) to give colorless resin (1.1 g, 83 %).

^{1}H NMR (400 MHz, DMSO-d$_6$) δ: 7.80 (d, *J* = 6.6 Hz, 2H), 7.40 (d, *J* = 7.0 Hz, 2H), 7.36 – 7.26 (m, 4H), 1.99 (q, *J* = 7.3 Hz, 4H), 0.20 (t, *J* = 7.3 Hz, 6H) ppm. ^{13}C NMR (101 MHz, DMSO-d$_6$): δ 149.25, 140.90, 127.16, 126.87, 122.83, 119.77, 55.51, 31.78, 8.35 ppm. Anal. calcd. for $C_{17}H_{18}$: C 91.84; H 8.16; found: C 91.66; H 8.44.

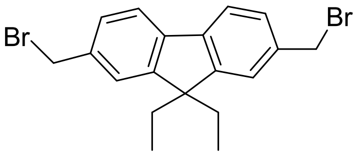

2,7-bis(bromomethyl)-9,9-diethyl-9*H*-fluorene (9)

A mixture of 9,9-diethyl-9*H*-fluorene (**8**) (0.75 g, 3.37 mmol), paraformaldehyde (0.53 g, 8.42 mmol) and 7 ml of HBr solution in acetic acid (33 %) was heated at 70 °C for 20 hours. After cooling to room temperature, the precipitate was collected and purified by column chromatography (acetone:*n*-hexane, 1:4), yielding 0.953 g (70 %) of white crystals; m.p.148-150°C.

^{1}H NMR (400 MHz, DMSO-d$_6$) δ: 7.80 (d, *J* = 7.7 Hz, 2H), 7.51 (s, 2H), 7.43 (d, *J* = 7.8 Hz, 2H), 4.80 (s, 4H), 2.03 – 1.99 (m, 4H), 0.22 (t, *J* = 7.5 Hz, 6H) ppm. ^{13}C NMR (101 MHz, DMSO) δ: 150.15, 140.51, 137.36, 128.54, 123.88, 120.22, 55.62, 39.52, 35.38, 31.66, 8.41 ppm. Anal. calcd for $C_{19}H_{20}Br_2$: C 55.91; H 4.94; found: C 55.68; H 4.81.

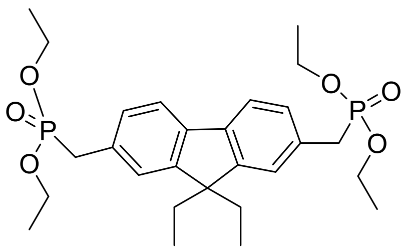

Tetraethyl [(9,9-diethyl-9*H*-fluorene-2,7-diyl)bis(methylene)]bis(phosphonate) (10)

2,7-bis(bromomethyl)-9,9-diethyl-9*H*-fluorene (**9**) (0.47 g, 1.15 mmol) was dissolved in triethyl phosphite (5.9 ml, 34.48 mmol) and the reaction mixture was refluxed overnight. After reaction completion (TLC, acetone: *n*-hexane 1:1), the triethyl phosphite was removed under reduced pressure. The crude product was purified by column chromatography (acetone: *n*-hexane 1:1) to give 0.375 g (63 %) of colorless resin.

^{1}H NMR (400 MHz, DMSO-d$_6$) δ: 7.70 (d, J = 7.7 Hz, 2H), 7.31 (s, 2H), 7.24 (d, J = 7.6 Hz, 2H), 3.92 (p, J = 7.2 Hz, 8H), 3.28 (d, J = 21.5 Hz, 4H), 1.97 (q, J = 6.8 Hz, 4H), 1.15 (t, J = 7.0 Hz, 12H), 0.21 (t, J = 7.1 Hz, 6H) ppm. ^{13}C NMR (101 MHz, DMSO-d$_6$) δ: 149.28, 149.27, 139.23, 131.07, 130.98, 128.61, 128.55, 124.33, 124.27, 119.42, 61.39, 61.32, 55.35, 33.31, 31.95, 16.20, 16.14, 8.31 ppm. Anal. calcd. for $C_{27}H_{40}O_6P_2$: C 62.06; H 7.72; found: C 61.91; H 7.50.

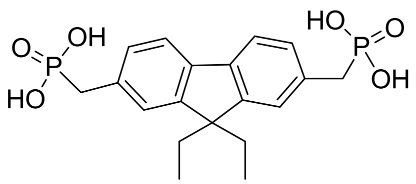

[(9,9-diethyl-9*H*-fluorene-2,7-diyl)bis(methylene)]diphosphonic acid (11)

Tetraethyl [(9,9-diethyl-9*H*-fluorene-2,7-diyl)bis(methylene)]bis(phosphonate) (**10**) (0.35 g, 0.67 mmol) was dissolved in anhydrous 1,4-dioxane (5 ml) followed by a dropwise addition of bromotrimethylsilane (1.7 ml, 13.32 mmol). The reaction mixture was stirred for 24 hours at 25 °C under argon atmosphere. After the termination of the reaction (TLC: acetone:*n*-hexane, 1:1), distilled water was added dropwise until white precipitate was formed and stirring continued for 72 hours. Precipitate was filtered off and washed with water to give 0.218 g (80 %) of white crystals; m.p. 242-243 °C.

^{1}H NMR (400 MHz, DMSO-d$_6$) δ: 8.07 (s, 4H), 7.65 (d, J = 7.7 Hz, 2H), 7.26 (s, 2H), 7.21 (d, J = 7.5 Hz, 2H), 3.02 (d, J = 21.4 Hz, 4H), 1.92 (d, J = 7.2 Hz, 4H), 0.30 (t, J = 7.0 Hz, 6H) ppm. ^{13}C NMR (101 MHz, DMSO-d$_6$): δ 149.36, 149.34, 138.75, 132.87, 132.78, 128.44, 128.38, 124.47, 124.41, 119.02, 55.14, 36.33, 35.02, 31.79, 8.66 ppm. Anal. calcd. for $C_{19}H_{24}O_6P_2$: C 55.61; H 5.90; found: C 55.85; H 5.72.

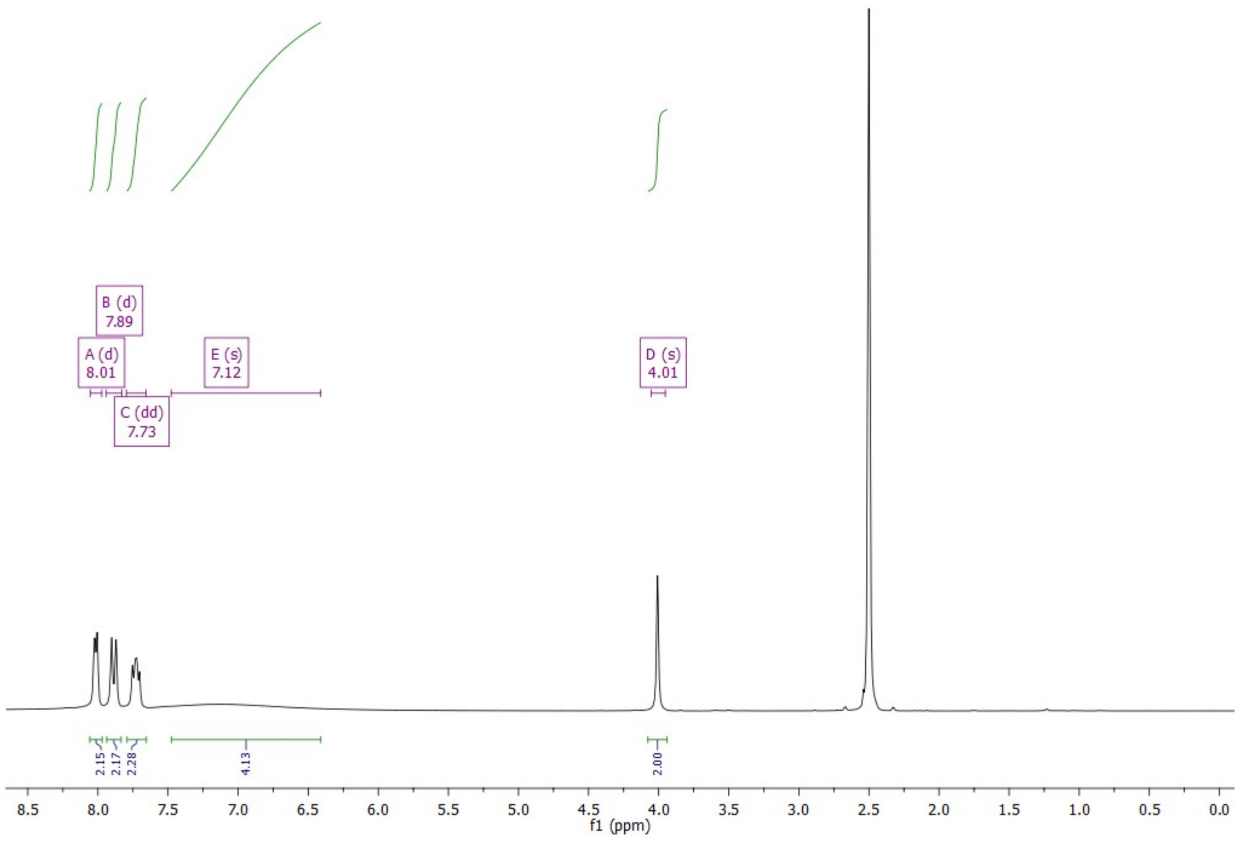

Figure S3. Fluorene-2,7-diphosphonic acid (**2**) ^{1}H NMR (400 MHz, DMSO-d_6, δ, ppm).

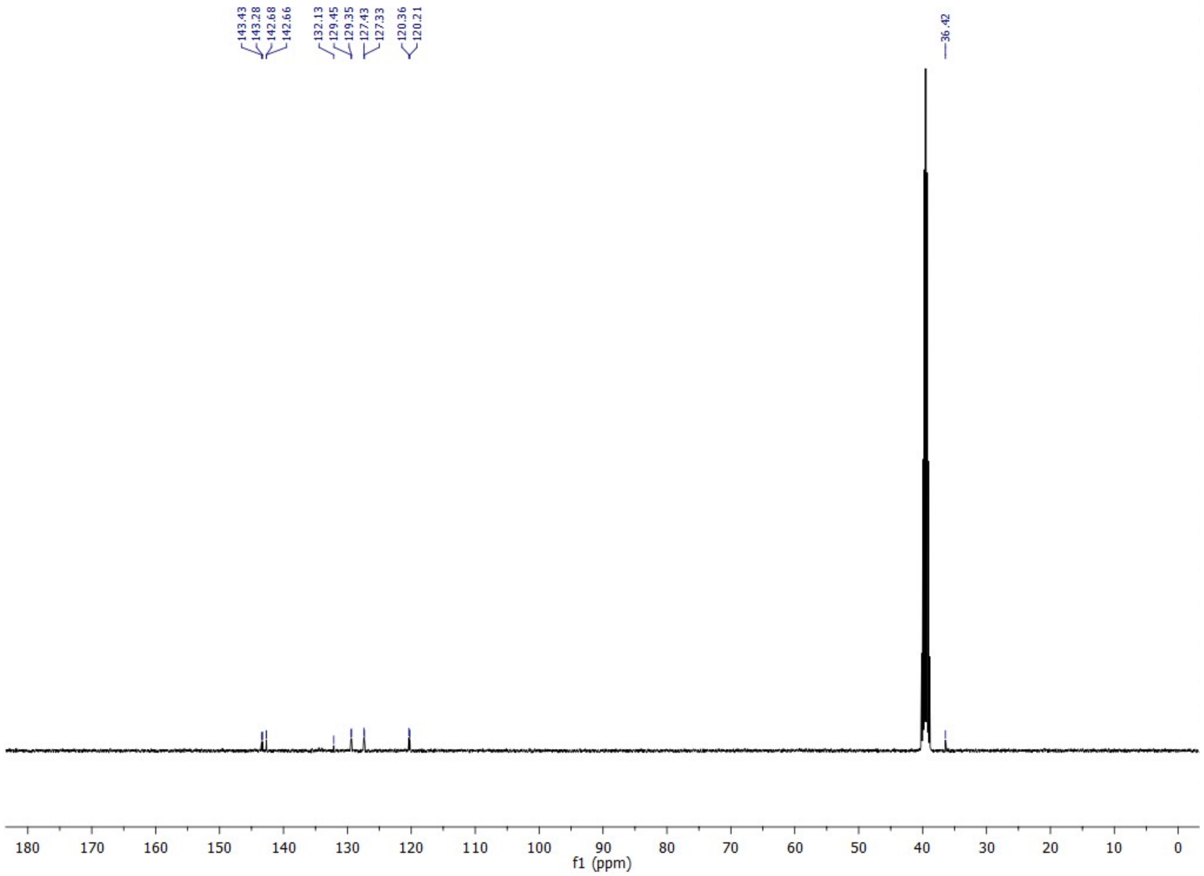

Figure S4. Fluorene-2,7-diphosphonic acid (**2**) ^{13}C NMR (101 MHz, DMSO-d_6, δ, ppm).

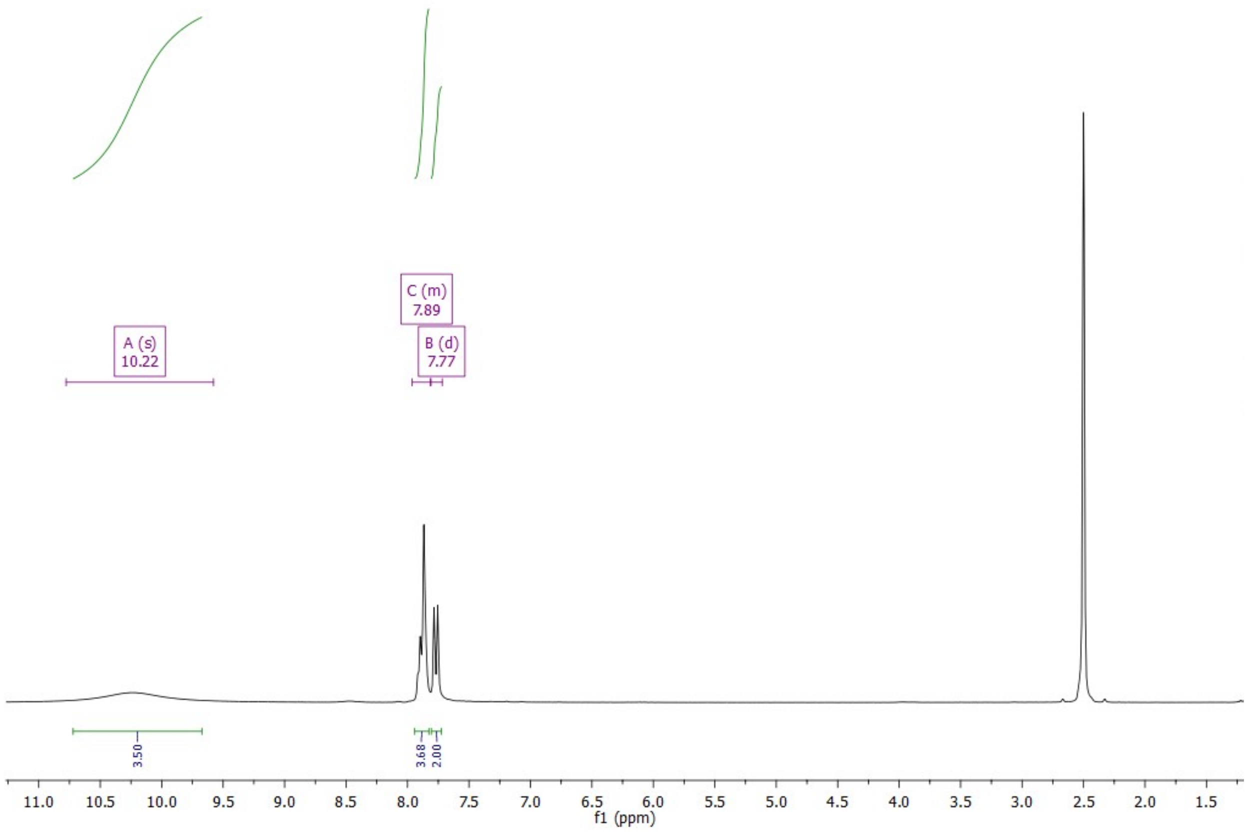

Figure S5. (9-oxo-9*H*-fluorene-2,7-diyl)diphosphonic acid (**4**) ^{1}H NMR (400 MHz, DMSO-d_6, δ, ppm).

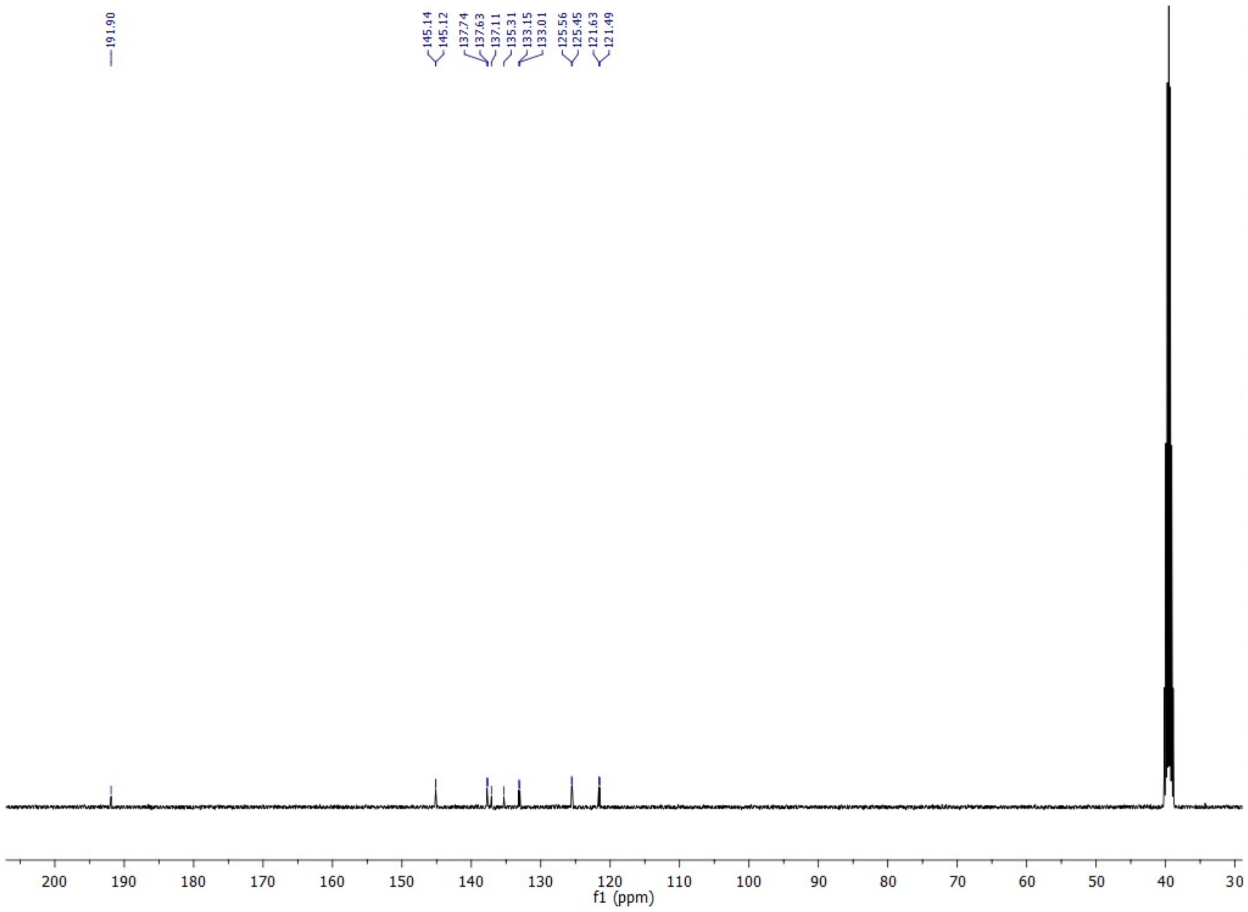

Figure S6. (9-oxo-9*H*-fluorene-2,7-diyl)diphosphonic acid (**4**) ^{13}C NMR (101 MHz, DMSO-d$_6$, δ, ppm).

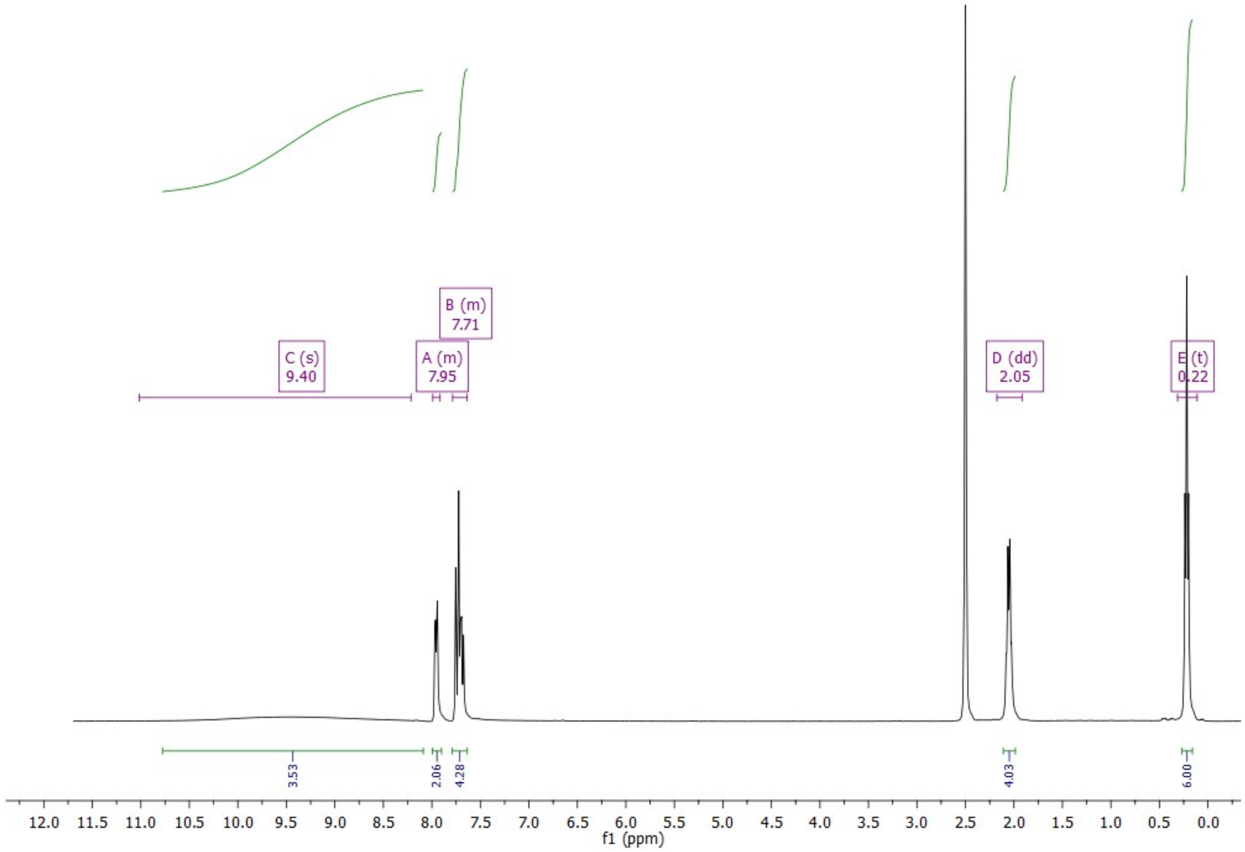

Figure S7. (9,9-diethyl-9*H*-fluorene-2,7-diyl)diphosphonic acid (**7**) ^{1}H NMR (400 MHz, DMSO-d_6, δ, ppm).

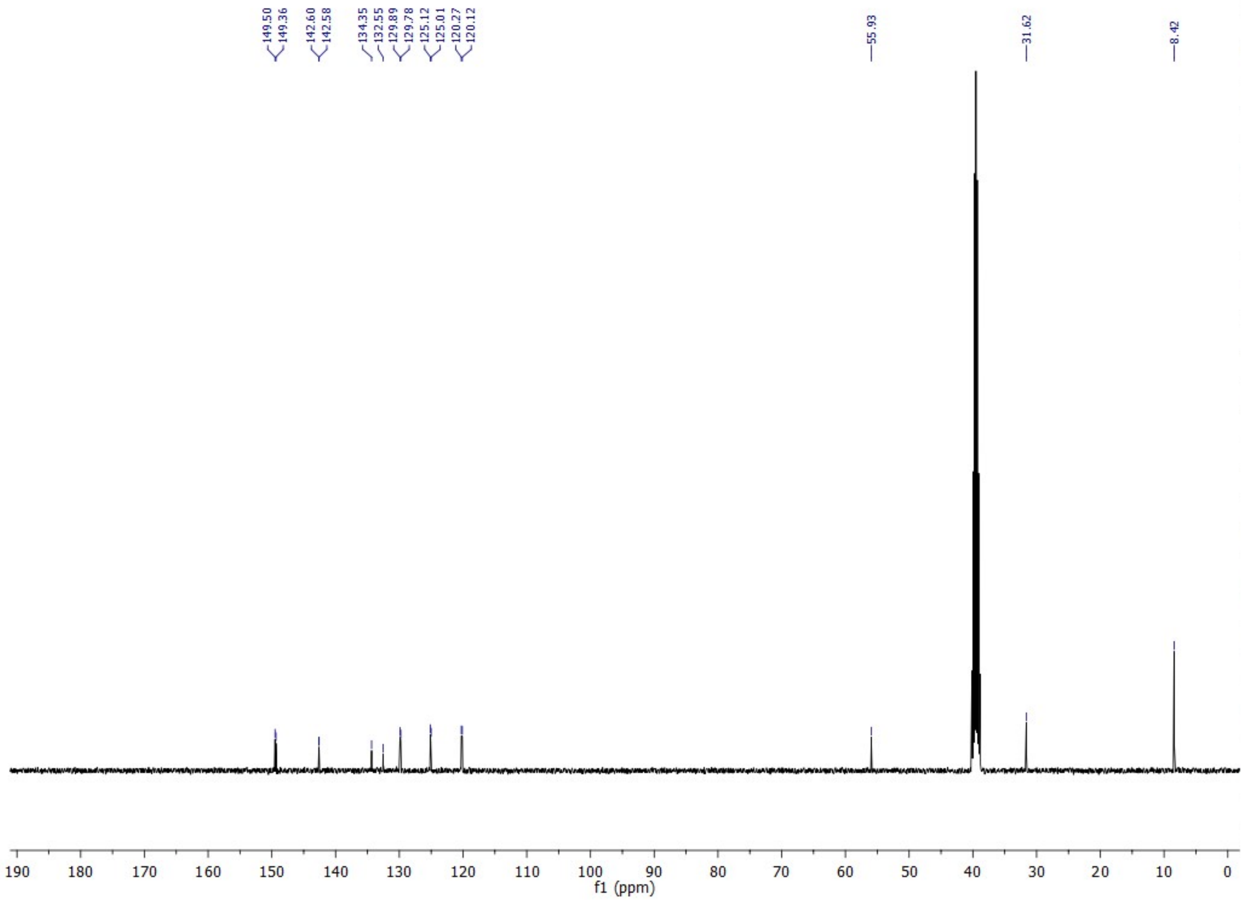

Figure S8. (9,9-diethyl-9*H*-fluorene-2,7-diyl)diphosphonic acid (**7**) ^{13}C NMR (101 MHz, DMSO-d_6, δ, ppm).

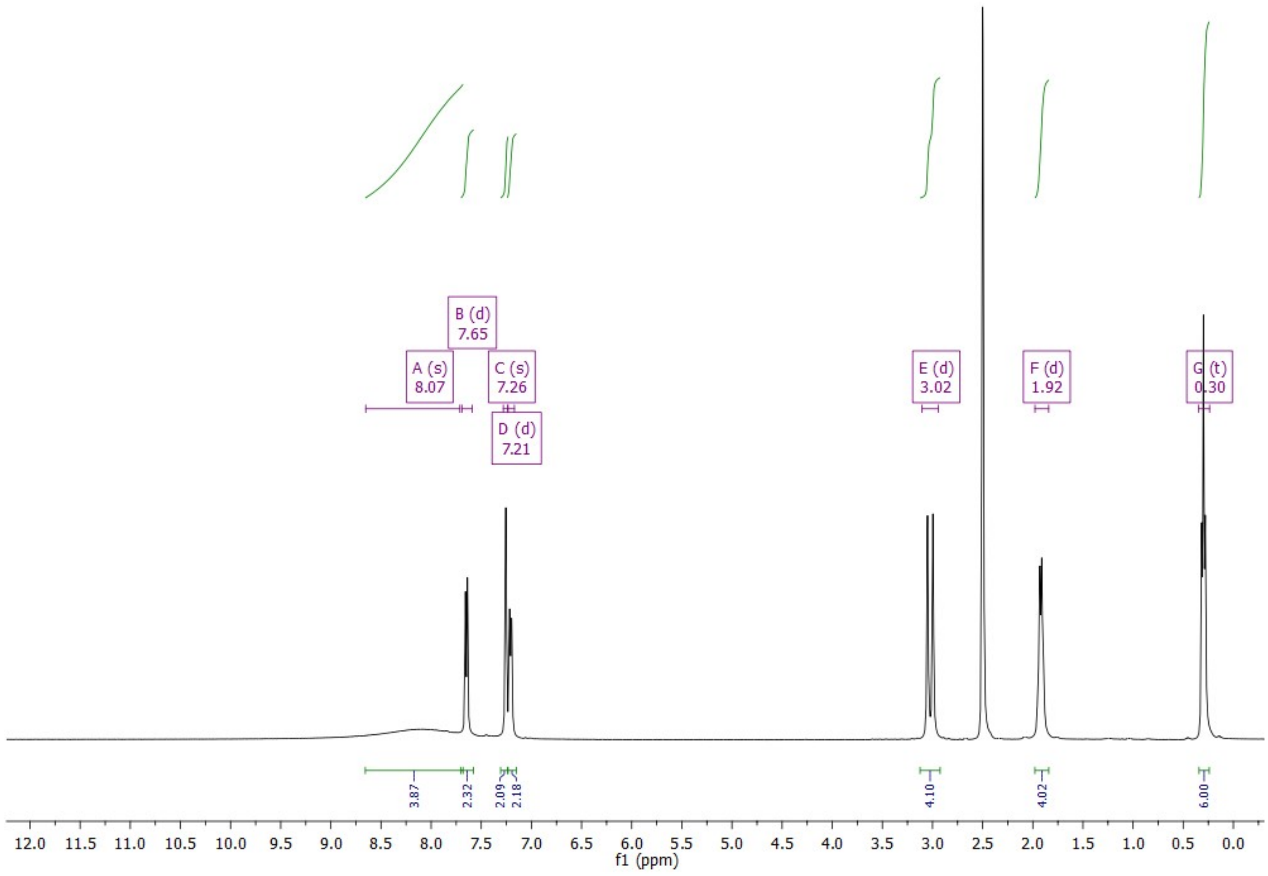

Figure S9. [(9,9-diethyl-9*H*-fluorene-2,7-diyl)bis(methylene)]diphosphonic acid (**11**) ^{1}H NMR (400 MHz, DMSO-d_6, δ, ppm).

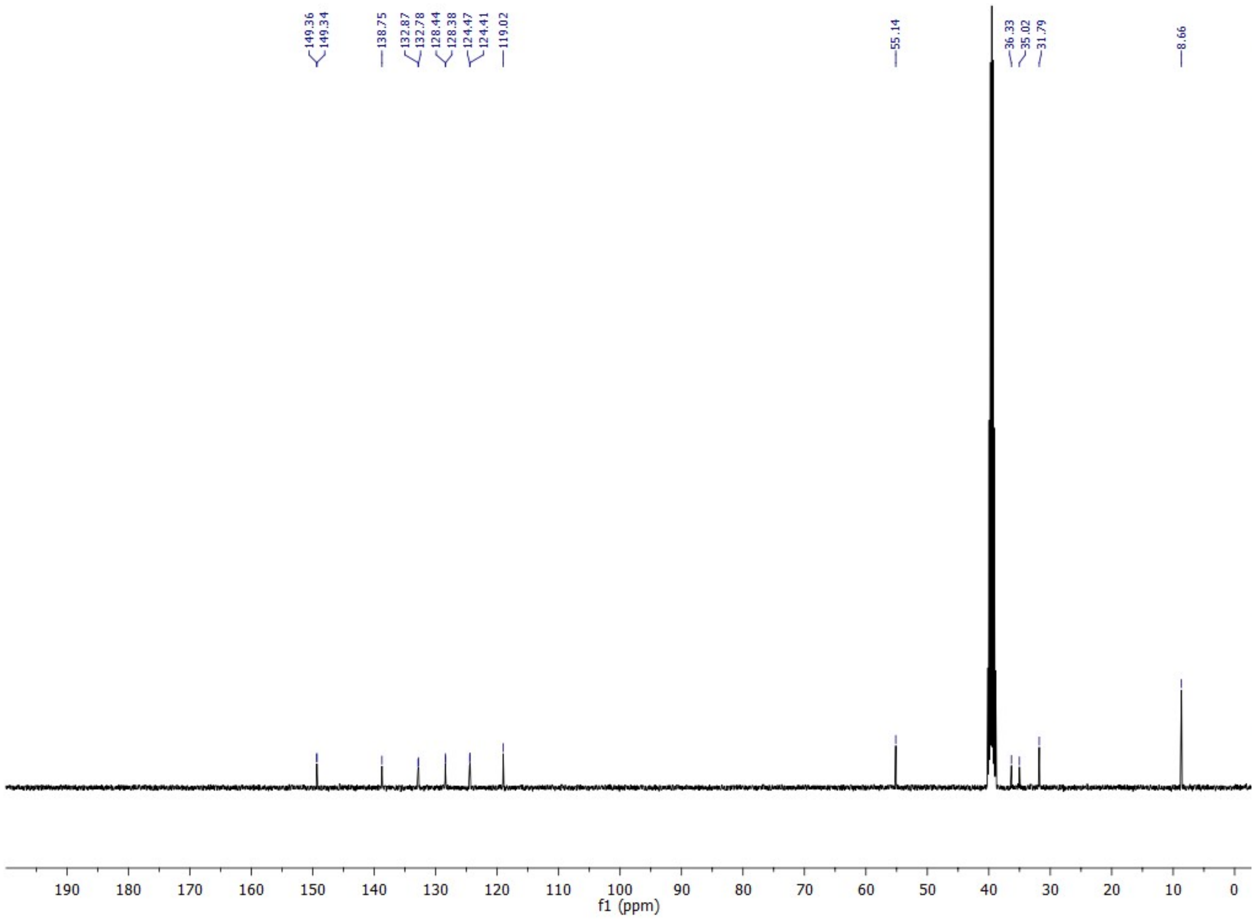

Figure S10. [(9,9-diethyl-9*H*-fluorene-2,7-diyl)bis(methylene)]diphosphonic acid (**11**) ^{13}C NMR (101 MHz, DMSO-d_6, δ, ppm).

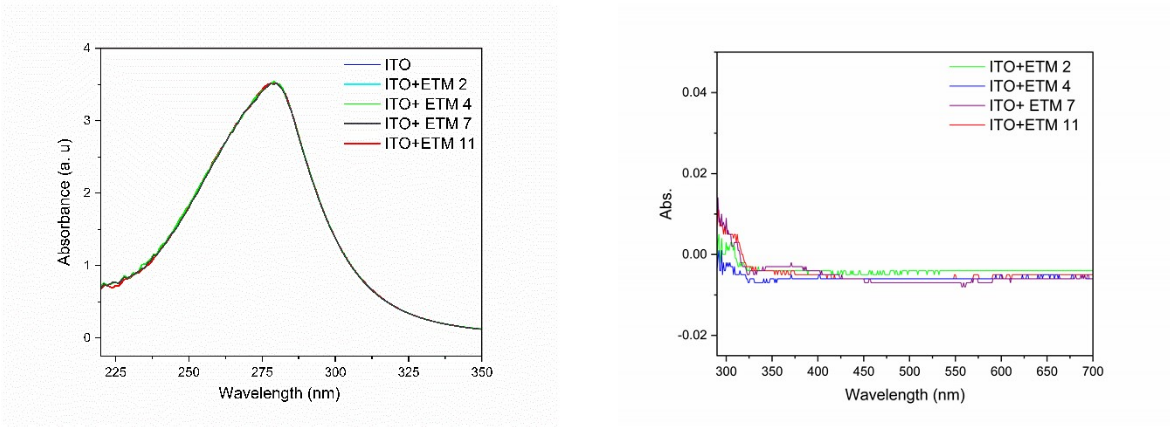

Figure S11. Absorption spectra of ITO coated different ETM SAM's: (a) baseline: Corning glass; (b) baseline: ITO.

Supplementary References

[1] R. Ahlrichs, M. Bär, M. Häser, H. Horn and C. Kölmel, Chem. Phys. Lett., 1989, 162, 165–169.

[2] C. Lee, W. Yang and R. G. Parr, Phys. Rev. B, 1988, 37, 785–789.

[3] A. D. Becke, J. Chem. Phys., 1993, 98, 5648–5652.

[4] A. Schäfer, H. Horn and R. Ahlrichs, J. Chem. Phys., 1992, 97, 2571–2577.

[5] F. Weigend and R. Ahlrichs, 2005, 3297–3305.

[6] N.G. Connelly, W. E. Geiger, Chemical Redox Agents for Organometallic Chemistry, Chem. Rev. 96 (2) (1996) 877–910. https://doi.org/10.1021/cr940053x.

[7] V.V. Paylishchuk, A.W. Addison, Conversion constants for redox potentials measured versus different reference electrodes in acetonitrile solutions at 25°C, Inorganica Chimica Acta 298 (1) (2000) 97–102. https://doi.org/10.1016/S0020-1693(99)00407-7.

[8] N. Juneja, S. Mandati, A. Katerski, N. Spalatu, S. Daskeviciute-Geguziene, A. Vembris, S. Karazhanov, V. Getautis, M. Krunks, I. Oja Acik, Sb_2S_3 solar cells with a cost-effective and dopant-free fluorene-based enamine as a hole transport material, Sustainable Energy Fuels 6 (2022) 3220–3229. https://doi.org/10.1039/D2SE00356B.

[9] J.S Eensalu, A. Katerski, E. Kärber, L. Weinhardt, M. Blum, C. Heske, W. Yang, I. Oja Acik, M. Krunks, Semitransparent Sb_2S_3 thin film solar cells by ultrasonic spray pyrolysis for use in solar windows, Beilstein J Nanotechnol. 10 (2019) 2396–2409. https://doi.org/10.3762/bjnano.10.230.

[10] R. Krautmann, N. Spalatu, R. Gunder, D. Abou-Ras, T. Unold, S. Schorr, M. Krunks, I. Oja Acik, Analysis of grain orientation and defects in Sb_2Se_3 solar cells fabricated by close-spaced sublimation, Solar Energy 225 (2021) 494–500. https://doi.org/10.1016/j.solener.2021.07.022.

[11] M. Koltsov, R. Krautmann, A. Katerski, N. Maticiuc, M. Krunks, I. Oja Acik, N. Spalatu, A post-deposition annealing approach for organic residues control in TiO_2 and its impact on Sb_2Se_3/TiO_2 device performance, Faraday Discuss, (2022) Accepted Manuscript. https://doi.org/10.1039/D2FD00064D.
